# Does Lateral Transmission Obscure Inheritance in Hunter-Gatherer Languages?

**DOI:** 10.1371/journal.pone.0025195

**Published:** 2011-09-27

**Authors:** Claire Bowern, Patience Epps, Russell Gray, Jane Hill, Keith Hunley, Patrick McConvell, Jason Zentz

**Affiliations:** 1 Department of Linguistics, Yale University, New Haven, Connecticut, United States of America; 2 Department of Linguistics, University of Texas at Austin, Austin, Texas, United States of America; 3 Department of Psychology, University of Auckland. Auckland, New Zealand; 4 School of Anthropology, University of Arizona, Tucson, Arizona, United States of America; 5 Department of Anthropology, University of New Mexico, Albuquerque, New Mexico, United States of America; 6 College of Arts and Social Sciences, Australian National University, Canberra, Australia; University of Pennsylvania, United States of America

## Abstract

In recent years, linguists have begun to increasingly rely on quantitative phylogenetic approaches to examine language evolution. Some linguists have questioned the suitability of phylogenetic approaches on the grounds that linguistic evolution is largely reticulate due to extensive lateral transmission, or borrowing, among languages. The problem may be particularly pronounced in hunter-gatherer languages, where the conventional wisdom among many linguists is that lexical borrowing rates are so high that tree building approaches cannot provide meaningful insights into evolutionary processes. However, this claim has never been systematically evaluated, in large part because suitable data were unavailable. In addition, little is known about the subsistence, demographic, ecological, and social factors that might mediate variation in rates of borrowing among languages. Here, we evaluate these claims with a large sample of hunter-gatherer languages from three regions around the world. In this study, a list of 204 basic vocabulary items was collected for 122 hunter-gatherer and small-scale cultivator languages from three ecologically diverse case study areas: northern Australia, northwest Amazonia, and California and the Great Basin. Words were rigorously coded for etymological (inheritance) status, and loan rates were calculated. Loan rate variability was examined with respect to language area, subsistence mode, and population size, density, and mobility; these results were then compared to the sample of 41 primarily agriculturalist languages in [1]. Though loan levels varied both within and among regions, they were generally low in all regions (mean 5.06%, median 2.49%, and SD 7.56), despite substantial demographic, ecological, and social variation. Amazonian levels were uniformly very low, with no language exhibiting more than 4%. Rates were low but more variable in the other two study regions, in part because of several outlier languages where rates of borrowing were especially high. High mobility, prestige asymmetries, and language shift may contribute to the high rates in these outliers. No support was found for claims that hunter-gatherer languages borrow more than agriculturalist languages. These results debunk the myth of high borrowing in hunter-gatherer languages and suggest that the evolution of these languages is governed by the same type of rules as those operating in large-scale agriculturalist speech communities. The results also show that local factors are likely to be more critical than general processes in determining high (or low) loan rates.

## Introduction

Darwin [2], [3] suggested that patterns of human biological and linguistic variation might correspond because of parallel tree-like evolution in isolated human groups. This tree analogy is often used by linguists to justify the use of phylogenetic methods to reconstruct the evolutionary process for a group of languages [4], [5]. However, some linguists have argued that lateral transmission or borrowing among languages is rife, making lexical phylogenetic methods inappropriate for reconstructing linguistic evolution [1], [6]–[8]. Lexical phylogenetic methods may be especially inappropriate for hunter-gatherer languages, where it has been suggested that rates of borrowing are particularly high [6], [9]. Such claims are not, however, based on broad-scale empirical work which measures loan rates.

Previous studies of borrowing are based on highly restricted samples from individual languages or small regions that lack standardized data sets, and results may therefore not be generalizable to other places and times. Some surveys [1] are standardized but contain too few languages to test for connections between rates of borrowing and demography. Here we redress these limitations by surveying loan rates in a large sample of hunter-gatherer (HG) and small-scale agriculturalist (AG) languages on three continents (Australia (AUS), North America (NAM), and South America (SAM); see Figure S1). These areas have adequate standard data sets, and they vary substantially with respect to the demographic, ecological, and social factors that are likely to affect borrowing.

### Hunter-gatherers and Language Change

The category ‘hunter-gatherer’ is defined principally with respect to food production – i.e., limited or no practice of agriculture. However, hunter-gatherer food production strategies vary in extent of cultivation, flora and fauna domestication, and food storage [10], [11]. They also vary with respect to social and demographic factors that can affect language change, such as sedentism, population size and density, settlement patterns, and social hierarchies, as well as in the degree of interaction with their neighbors and complexity of their social network organization [12]. Such variation may occur both across groups and over time; this is particularly the case in the SAM and NAM regions, where some groups have shifted back and forth between subsistence foci [13].

Social and demographic factors influence language change in both HG and non-HG groups via their effects on the rates and types of linguistic items that are borrowed [9], [14]. Because HG groups often have different demographic profiles from AG groups, such as smaller population sizes and tighter in-group social network structures [10], [12], it might be expected that changes conditioned by these factors would apply to HG and AG groups unequally. Phenomena proposed to guide processes of borrowing have included a language's structural profile [15] and, in certain cases, cultural constraints that severely penalize language mixing [6], [16]. In the former case, the amount of morphology that words contain has been linked to borrowability [17], [18].

Dixon [6] argues that roughly equal socio-economic status between HG groups should facilitate transfer in both directions. Dixon's claim is not specifically about hunter-gatherers, but about groups in “equilibrium,” particularly groups in Australia. However, because of the framing of his model and the treatment of agriculture and organized warfare as punctuation events, Dixon's arguments apply particularly to hunter-gatherers. Nettle [9] appeals to general processes which he argues apply specifically to Australia, but which result from demographics which are characteristic of hunter-gatherer groups. Additionally, where relations exist between hunter-gatherers and their agriculturalist neighbors, the tendency for the hunter-gatherers to be perceived as having relatively low social status might lead to greater borrowing between these groups [19]. All these works lead to a picture that languages spoken by HG communities are different from other languages.

None of the claims for higher HG lexical borrowing rates have been investigated systematically across a variety of language families. Here, we examine the dynamics of loans in 122 HG and AG languages from AUS, NAM and SAM. The sample contains the largest collection of hunter-gatherer lexical etymologies to date. We compare the results obtained from this sample with a set of AG and urban (URB) languages from the World Loanword Database project [1], [20] (hereafter WOLD). The present study is the first to empirically establish overall loan rates in basic vocabulary for such a broad sample of languages, and to examine the impact of size of area occupied by the language group, subsistence mode, population size, density, and mobility on rates of borrowing. We find that loan levels vary both within and among regions. We find low levels of borrowing in an array of languages with different demographic, ecological, and social conditions. The causes of especially low levels of borrowing in SAM, and the rare cases of exceptionally high borrowing, are explored. There is little support for claims that HG languages are significantly different from AG languages. instead, local social and historical factors prevail. Claims that tree-building is impossible in these language due to rates of loans are thus incorrect; rates overall are an order of magnitude smaller than the loan rates which lead to loss of phylogenetic signal using lexical data [21].

## Results

### General levels

**Figure 1:**
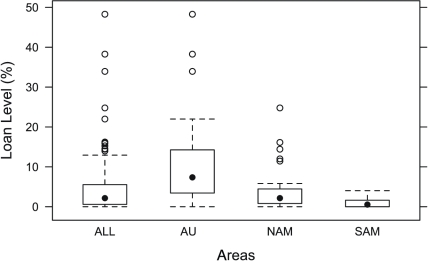
Loan figures aggregated and by region. Box-and-whiskers plot of loan levels aggregated for all regions (ALL) individually for the case study areas (AUS = Australia; NAM = California and the Great Basin area of North America; SAM = Amazonian region of South America).

**Figure 2:**
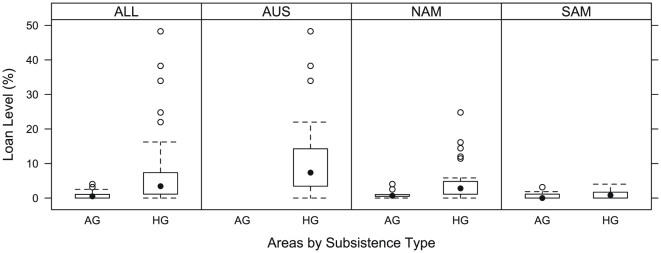
Subsistence patterns. Box-and-whiskers plot of Case Study Area loan data, split by subsistence type (HG = hunter-gatherers, AG = agriculturalists).

Figure 1 gives the loan figures by region for HG and AG languages from this sample (full details are in Tables S1 and S2). The mean borrowing rate for the sample is 5.06%, with median 2.49%, and SD of 7.56. The lowest rate is 0, signifying no loans in the wordlist sample, and the highest is 48%. These figures are lower than those reported in the World Loanword Database (WOLD) [20], where the mean on an equivalent wordlist is 10.24% (median 5.3%, SD 11.02, range 0%–45%). A recent study of loans in Indo-European languages [22] found an average loan rate of 8%. Figure 2 splits the regions by subsistence type, and Figure S2 presents results by density, population size, mobility, and exogamy.

Table S2 also presents information regarding the comparison of loan levels to other sources of vocabulary within the wordlist. On average, 28% of the words in the sample had no clear etymology within the language. There was no significant correlation between the number of loans in a language and the number of unique items in that language (r = −0.025; P = 0.722); nor was there any significant correlation between whether a given lexical item in the wordlist was coded as loaned or unique (r = 0.097; P = 0.168). This indicates that detection rates for loans did not deviate significantly across the sample, and that unique items are not simply unidentified loans.

### Amazonia (SAM)

The SAM sample draws from ten distinct language families, reflecting the high linguistic diversity of the northwest Amazon. Borrowing rates are uniformly low in these languages, with no language exhibiting more than 4% loans in its basic vocabulary, and most ranging between 1–2%. The rate is low in the region despite substantial variation in the level of contact between groups, social status, subsistence modes, and demographic situations.

There is ample evidence for variation in the type and intensity of contact among different groups in the region. For example, speakers of Huaorani (a language isolate located on the Ecuador-Peru border) have historically maintained minimal interaction with neighboring groups. At the opposite end of the spectrum are the multilingual peoples of the Vaupés region in eastern Colombia and northwest Brazil. For the more horticulturalist Vaupés peoples (East Tukanoans and some Arawaks), this multilingualism derives principally from their practice of obligatory marriage across language groups, known as linguistic exogamy. For the foraging peoples of the region (Nadahup and Kakua), widespread but unreciprocated bilingualism is an outcome of their intensive ‘client’ relationship with their horticulturalist neighbors [16], [23]. Among the Vaupés peoples, cultural attitudes condemning language mixing impede lexical borrowing and code-switching, but do not appear to be a significant obstacle to grammatical diffusion [16], [23], [24]. Neither food production strategies nor exogamy are significant predictors of loan rates in this area (p = 0.668 and p = 0.576 respectively).

Loan rates are low regardless of social status in SAM. For example, among Vaupés agriculturalists, who are of relatively high status compared to the hunter-gatherers, Tukanoan groups exhibit only 0–1% loans, and Arawak Tariana has under 2%, despite the fact that its speakers are currently shifting to Tukano. The Vaupés hunter-gatherers all have relatively low social status coupled with intensive interaction with horticulturalists. They also exhibit low borrowing, e.g., under 2% among the Nadahup languages, mostly from Tukanoan, and approximately 4% for the Kakua of the Kakua-Nukak group, many from its hunter-gatherer Nadahup neighbors. The Nukak language, also of the Kakua-Nukak group, but spoken outside the Vaupés, has about 2% loanwords, also mostly from Tukanoan languages. Other northwest Amazonian languages show similarly low rates of borrowing, despite a range of different contact situations. No clear loans were identified for any of the Yanomami languages, for example, despite their engagement with Carib and neighboring peoples.

Population size was significant as a factor in loan rates (p = 0.015). Small population size was predictive of higher loan rates, though due to globally low rates, the difference is only 1%. Density of settlement was not significant (p = 0.247).

### California and the Great Basin (NAM)

Aboriginal California exhibits a high level of linguistic diversity, with more than 100 languages in 7 major lineages [25], [26]. Many of the language communities were small, and there were intricate relationships among them, including shared ceremonial activity, trade, and intermarriage, which yielded extensive multilingualism. In most California languages loan rates are low in spite of this intensive contact. For instance, Takic (Uto-Aztecan) and Yuman languages in southern California all exhibit very low rates of loans in our sample of basic vocabulary, although Hinton [27] reported phonological convergence among them. However, our sample confirmed a few cases of extensive linguistic interchange. The Yukian language Wappo exhibits so much influence from unrelated languages of the region that its genetic affiliation with Yukian has been controversial [26]. Wappo in our sample exhibits a loan rate in basic vocabulary of 14.3%. Callaghan [28] documented striking phonological convergence of Lake Miwok to the neighboring Pomoan languages, and the Wintun language Patwin, and Lake Miwok in our sample exhibits a loan rate of 11.4%. These rates are relatively high in the North American context, where the maximum loan figure is 24% and 41 of the 47 languages in the sample have loan levels under 10%.

Mobility (p = 0.048), population size (p = 0.045), and population density (p = 0.046) were significant factors in the NAM area. Mobile populations and populations with low density of settlement had significantly lower rates, while those with small populations were predictive of higher rates. With respect to food production, HG groups had mean 4.4% loans, median 2.8%, SD 5.23, while agriculturalists had consistently lower figures (mean 1.1%, median 0.66%, SD 1.26; p = 0.051). No groups preferred linguistic exogamy.

### Australia (AUS)

High borrowing related to language contact has featured prominently in historical analyses of Australian languages. Especially influential has been the work of Heath [29], [30], but others have reported high levels of borrowing in other parts of the country [31], [32]. High borrowing is reported for the sole example of an Australian language in WOLD [32], where the Ngumpin-Yapa language Gurindji has borrowed almost 50% of list items. Borrowing is also high in Gurindji in our sample, at 48% in the basic vocabulary, but this high level is atypical of the Australian languages in our large sample.

Despite intensive contact, the number of loan items in basic vocabulary for most languages is smaller than the cases previously cited, with a mean of 9.4% (median 5.54%, SD 11.01). The data reveal considerable variation in loan rates, even among languages that had extensive interaction with their neighbors. The results range from 0% loans to 48%. The highest figures (above 30%) are found in a few languages in the Victoria River District. A second small group of languages has approximately 25% of their basic vocabulary borrowed. 35 languages have figures of 10% or less, and another 10 have loan levels less than 20%. The languages with highest loan figures are Gurindji (49%), Mudburra (38%) and Gooniyandi (33%). These three languages were clear outliers (Fig. 1, Table S2). When these outliers were eliminated, the mean number of loans in the AUS sample dropped to 6.61%, in line with values reported in other regions.

The AUS sample also includes Yolngu languages from Eastern Arnhem Land, which have been prominent in claims regarding the frequency of Australian lexical borrowing [33]. Heath found high rates of shared lexicon between Ngandi and Ritharrngu. Our sample showed Ritharrngu's borrowing rate at 22%, with 25 loans coming from Ngandi. Others loans include 2 from English, 7 from Wubuy, and 2 from Djambarrpuyngu. Ngandi loan rates are lower, but 19 of the 21 loans in the sample come from Ritharrngu. *A-barra* ‘wind’ is from the Austronesian language Makassar, though possibly via Ritharrngu or another Yolngu language, and *dhaku* ‘small’ is from Rembarrnga. Symmetrical borrowing has increased the percentage of ‘shared’ vocabulary across these languages, which straddle the Pama-Nyungan–Non-Pama-Nyungan border. Note, however, that in no cases here does the presence of high levels of symmetrical borrowing prevent recovery of family affiliations, a point also noted by Heath [30].

**Figure 3:**
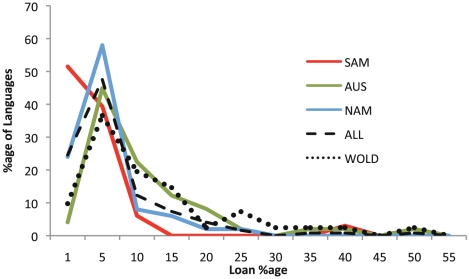
Loan levels across the regions. Line plot of loan levels in Australia (AUS), North America (NAM) and South America (SAM) compared to the aggregate sample (ALL) and the languages from Haspelmath and Tadmor (2009) [1], showing an overall similarity in the distribution of loan levels by language across regions and sample type.

In summary, the two areas of Australia that have received greatest attention in the literature for loans are revealed with a more representative language sample to be the most atypical. Figure 3 plots the distribution of Australian loan levels versus those of other languages outside the country (the SAM and NAM case study regions; our sample combined, and the WOLD dataset). Our findings are in agreement with another study of rates in Australian languages; Alpher and Nash [34] examined loans in 14 languages of the Cape York region, and found that rates of lexical replacement by borrowing in this area were maximally between 10% and 24%, within (though toward the upper end) of the variation found in the current survey, and well below the rates claimed by Dixon [6].”

Australian groups are all traditionally hunter-gatherers, so subsistence levels were not compared. All lived in mobile bands but the degree of mobility varied greatly between a seasonal round mainly within clan estate territories over relatively short distances of 50 km or less in the regions with more natural resources, to long-distance nomadic travel in the arid zones. Exogamy between language groups was not obligatory in most areas, but residential bands tended to include speakers of more than one language. Small populations are more likely to have higher loan levels than medium or large ones (p = 0.015), and languages spoken by groups with low population density are more likely to have higher loans than densely populated groups (p = 0.023); this is the reverse of the NAM case study, were low density populations had lower loans. Moreover, once outliers are removed in the AUS sample, small group size becomes less significant (p = 0.090) and low population density is non-significant (p = 0.560). Mobility is not significant (p = 0.208). Exogamy is also significant (p = 0.039).

### Differences between case study areas

In all areas, little basic vocabulary is borrowed, despite substantial variation across the regions in the level of contact between groups, social status, subsistence modes, ecology, and demographic situations. In SAM, no language borrowed more than 10% of the sample vocabulary (all were in fact much lower). In NAM, 90% of the languages borrowed 10% or less, while in AUS 62% had 10% or fewer loans. SAM can be characterized as uniquely low-borrowing, with 58% of the languages borrowing either 0 or 1 item. Only 26% of languages in NAM and 7.5% of languages in AUS borrowed at such low rates. Thus, while in all areas borrowings are low, in SAM they are markedly so. Figure 3 plots the distribution of loans for each of the case study areas, the WOLD dataset, and the aggregated sample. The difference between all case study regions was significant (p<0.01).

### Hunter-gatherers vs. agriculturalists

Mean borrowing rates for all HGs in our sample was 6.38%, median 3.44%, SD 8.85; for agriculturalists the figures are mean of 5.15%, median 1.95%, SD 8.1. Differences between HG and AG groups are significant overall but skewed by the AUS area (which has no AG groups); within areas there is no significant difference. Thus individual area variation is more important than any general tendencies of HG or AG languages.

### Population size, density, and mobility

Our results reveal no association between rates of lexical borrowing and numbers of speakers (p = 0.735). Most languages in the HG sample have current speaker populations under 5,000 individuals. Three of the SAM groups are considerably larger, and we note that many indigenous groups within our sample have experienced profound demographic changes since European arrival that are not well documented. Within areas, however, small populations were predictive of higher loan rates. Density is not significant (p = 0.600) overall, and note that while low population density was a significant factor in both NAM and AUS, in the former it correlated with low loan levels, while in the latter low density populations had higher loan levels. Mobility is significant overall (p = 0.010), and within NAM and SAM, though not within AUS. Note that in SAM and NAM, mobile populations showed opposite trends, with lower loan levels in NAM but higher ones in SAM.

### Exogamy

Linguistic exogamy overall was highly significant (p = 0.001) and associated with high borrowing in the total sample. This implies that exogamy is a likely factor in driving loans in the absence of other social prohibitions on language mixing (as are found in the SAM case study, where exogamy was not significant).

### WOLD dataset

Borrowing in our AUS, NAM, and SAM sample is lower than in the WOLD dataset, where the mean number of loanwords in the 204-word sample under consideration was 10.24% (median 5.3%, SD 11.02; the difference is significant (p 0.001). The WOLD dataset is smaller than the dataset constructed for this paper, containing only 41 opportunistically sampled languages from across the world. Though the dataset samples a wide range of languages, it does not control for differences in demography; moreover the authors [1] report that their sample is likely to overestimate loan averages because of a tendency to sample languages independently known in the literature for relatively high rates of borrowing.

## Discussion

We hypothesize that the very low rates in SAM may be indicative of an association between language and group identity that is relatively strong compared to many other parts of the world, and pertains widely within Amazonia. Such an association is particularly salient in the Vaupés region, where low loan rates are tied to the practice of linguistic exogamy; see above and [35]), but our results suggest that the tendency to keep languages distinct is more widespread in the region. The AUS sample has higher loan rates than the other two areas, and also shows considerably more variation. However, only two cases (Gurindji and Mudburra) approach the levels of borrowing claimed to be the norm for the continent [6]. The extreme rates in some AUS languages are partially accounted for by a few loans from English, including ‘roof’, ‘rope’, and ‘work’, though English loans do not account for the highest rates, and English loans are all but absent from Gurindji, the language with the highest loan rate in the sample.

### Directions of borrowing

Because of the low rates of borrowing among so many of the case study languages, a detailed quantitative study of loan sources was not possible. However, qualitative comments can be made. The case study areas contain cases of both symmetrical and asymmetrical borrowing. The highest borrower in the sample, Gurindji, is heavily asymmetric. The direction of flow of loans is predominantly into Gurindji from its northern neighbors; some vocabulary items that went in the opposite direction can be identified, but these are few. Between Mudburra and its eastern neighbor Jingulu, however, the flow seems to be bidirectional. The same is also true for other pairs in AUS, such as Yawuru and Karajarri.

In NAM, both types of borrowing are identifiable. In Southern California, the mostly AG Yuman and Uto-Aztecan languages exhibit symmetrical borrowing from one another (at very low levels). In contrast, in the north, the Yukian language Wappo has loans from Pomoan and Wintun languages, but is not a donor into Wintun.

In the SAM sample, borrowing is predominantly asymmetric; Arawak languages are frequent sources of loans into other languages, although this directionality appears to be reversed in the Vaupés, where Arawak Tariana has experienced profound contact with Tukanoan languages. It is also reversed in southwest Colombia, where Arawak Resígaro has borrowed from Bora; note that these languages are all AG. HG languages in contact with AG languages are predominantly recipients of loans, both in the Vaupés, where the HG languages Hup, Yuhup, and Kakua have borrowed from AG Tukanoan, and also in other cases, e.g. HG Nadëb from AG Arawak. Borrowing between HG languages is attested in the case of Hup (Nadahup) and Kakua (Kakua-Nukak), and appears to be predominantly asymmetric (Hup into Kakua).

### Causes of high borrowing

Since the norm is low borrowing (below 10%), the outliers with high rates (>30%) require an explanation. Possible causes include mobility, intensity of language contact, asymmetries in social hierarchies between groups, and comparative differences in population sizes and densities.

There is equivocal support for the idea that mobility is associated with exceptionally high borrowing. On the one hand, in the NAM sample, there appear to be no differences in loan frequency between mobile Uto-Aztecan languages of the Great Basin and comparatively sedentary groups in California, including the Southern California Uto-Aztecan languages. In the Uto-Aztecan group, the highest loan frequencies appear in Tübatulabal and Bankalachi-Toloim, spoken in relatively sedentary communities (with some seasonal mobility). There is also no difference in the SAM area. Within AUS, however, the highest borrowing languages are all spoken by mobile populations; seasonal and sedentary loan levels in AUS are comparable to the other case study areas. In the WOLD sample, some of the highest loan figures are also found in mobile populations (such as Selice Romani [36]). Thus there would appear to be some support for mobility being a factor in exceptionally high borrowing cases.

High intensity of language contact does not by itself explain high rates of borrowing. All the languages in AUS, for example, were in contact with their neighbors and participated in trade networks [37], yet only a few show extreme borrowing rates. Several SAM and AUS groups are linguistically exogamous, but this practice is not correlated with loan levels within the case study areas which have differences in exogamy. For example, in SAM, rates of loans are universally low among both linguistically endogamous and exogamous communities. In AUS, both exhibit variable rates.

Another possible factor that mediates variation in loan rates is prestige asymmetries among local groups. Though this factor is invoked to explain variation in a number of languages in the WOLD sample [1], for example loans into Saami from Russian, into Berber from several Arabic varieties, and from numerous languages into Selice Romani [36], it is impossible to quantify. Long-standing exposure to literacy is associated with high borrowing in the WOLD datasets; all the high-borrowing languages (except Gurindji [32]) feature loans from ancient literary languages, such as Thai and Indonesian from Sanskrit (the latter also from Arabic). Borrowing from literary languages is not a factor in our sample.

Language shift could explain two cases of high borrowing. In Australia, there is evidence that Gurindji and Mudburra have acquired speakers. The Eastern Ngumpin languages bulge north into the riverine zone from the desert to the south, and separate the two discontinuous branches of the Mirndi family. These languages probably spread north into the Victoria River Basin, adopting a great deal of environmental vocabulary in the process. McConvell [32] proposes that this process involved past language shift to Gurindji, with uptake of both substrate and adstrate vocabulary.

Bankalachi Toloim (NAM) shows heavy loans from Yokuts. Evidence [38] suggests that the consultants who provided these data were from a speech community that had been in the process of shifting to a variety of Yokuts. Thus while language shift may be a factor in high loan rates, this requires further work. Note, for example, that the two shift cases in the case study are opposites, with Gurindji acquiring loans while gaining speakers, and Bankalachi Toloim in the process of losing speakers. Moreover, language shift is also ongoing in Tariana (SAM) but has not resulted in heavy lexical borrowing [23].

### Implications for Phylogenetic Reconstruction

The criticism of non-treelike linguistic evolution in HG groups, even in cases where it is shown empirically to be valid, does not prevent the application of other methods used by biologists to examine evolutionary process, such as network analysis [39]. These methods provide information about the magnitude and pattern of exchange between groups and may be productively used in concert with phylogenetic methods [40]. These methods are likely to be particularly valuable in the study of genetic and linguistic coevolution.

While it is important to identify the occasional aberrant cases of high borrowing, our results support the idea that lexical evolution is largely tree-like, and justify the continued application of quantitative phylogenetic methods to examine linguistic evolution at the level of the lexicon (see also [22]). As is the case with biological evolution, it will be important to test the fit of trees produced by these methods to the data used to reconstruct them. However, one advantage linguists have over biologists is that they can use the methods we have described to identify borrowed lexical items and remove them from the dataset [41]. For this reason, it has been proposed that, in cases of short to medium time depth (e.g., hundreds to several thousand years), linguistic data are superior to genetic data for reconstructing human prehistory [5], [42].

Finally, this work also demonstrates the utility of linguistic tree building using basic vocabulary. Linguists have sometimes argued that trees constructed from lexical items alone are too subject to interference from loans to show accurate histories [43]. While in a few areas, loan levels approach or exceed the rates which are likely to interfere with phylogenetic signal [21], 96% of the languages in the sample had loans well below the threshold at which we might expect interference.

### Conclusions

In summary, basic loan levels in languages are usually low, no matter what the factors. Certain social situations may lead to either abnormally low levels, as in SAM, or very high levels. High levels of loans can be the result of several different factors, including language shift and access to writing. There is also some evidence that mobile populations have higher average rates of borrowing. No evidence was found for a difference in loan rates between HG and AG groups within the case study regions, suggesting that the social differences between HG and AG languages that resulted from the Neolithic revolution have not been as important for this area of language change as has been claimed.

## Materials and Methods

### The Languages and Language areas

Languages spoken by hunter-gatherers and agriculturalists from Australia, North America (Southern California and the Great Basin), and South America (Amazonia) were examined and coded for etymology (Table 1). Australian indigenous languages were traditionally spoken only by hunter-gatherers [37]. Coding of this type requires specialist knowledge of the languages; thus focal areas for case studies are those for which the authors have the requisite knowledge, where accurate data were available, and where the genealogical affiliation of the languages is reasonably well established. Accordingly, we focus our sample on these three regions, although languages spoken by hunter-gatherer groups occur more widely, e.g. in southeast Asia and southern Africa. We note that we have sampled approximately 20% of the extant hunter-gatherer languages still spoken, distributed across three independent geographic regions, which is already many times more broad than previous loan surveys.

**Table 1:** Summary of languages by survey regions.

Area	Families	HG	Non-HG	total
Australia	6	31	0	31
North America	6	36	10	46
South America (Amazonia)	8	14	13	27

See Tables S1 and S2 for further information.

While the quality of data varies considerably within regions, attempts were made to use the most complete and accurate sources. Source information for the languages is available in the supporting documentation. For all case study areas, languages with good documentation (and for which the surrounding languages were well-documented) were prioritized, in order to minimize possible effects of data quality on the ability to identify loans. We recognize that there are cases where loan identification is difficult [44]; however, steps were taken to minimize such problems in this dataset. Languages were sampled from a variety of families, many of which are not closely related; this makes loan identification more straightforward, since loans (especially recent ones) tend to be phonologically similar, while inherited items are more distinct. Second, the areas are those in which the authors have the requisite specialist knowledge of the languages.

The 49 languages for consideration in the AUS case study are some well-attested northernmost subgroups of the Pama-Nyungan family, along with their non-Pama-Nyungan neighbors from the Kimberley region, Victoria River district, and Arnhem Land. The large time depth between those groups makes loans easily identifiable; furthermore, there has been previous historical work on the sound changes in the area, which allows loans and inherited items to be identified with some certainty [45]. All these languages are spoken by HG groups, but the groups vary in mobility, population size, density, extent of exogamy, and patterns of multilingualism.

The NAM sample includes 46 languages of California and the Great Basin. Languages north of the Sacramento Valley, including all of the Athapaskan languages and the two varieties of Algic spoken in California, were not included. Additional sources for loan identification were consulted; these are listed in the supporting materials. Both comparativist and arealist studies have a 100-year-long history in the area [46]. Where dictionaries were not available, lexical material was retrieved from grammatical studies and from archived field notes [38].

The SAM sample draws on 27 languages of the northwest Amazon, straddling Colombia, Peru, Ecuador, Brazil, and Venezuela. Just under half are spoken by peoples with a relatively strong emphasis on hunting/gathering. While comparative studies of these language groups are for the most part still in their infancy [26], [47], this work was informed by state-of-the-art internal classifications (see Table S2 for references). The lexical items in the sample languages were systematically compared with vocabulary from 72 other South American languages (almost all from the northwest Amazon region), corresponding to 18 language families and 13 isolates; these and the sources consulted are listed in Table S2.

The 43-language WOLD sample includes 12 languages from Eurasia, 8 from Africa, 10 from Southeast Asia and the Pacific, 4 from Central America and 6 from South America. These languages are predominantly spoken by agriculturalists (n = 23) or are urban, national languages (n = 11). Only 7 languages in the WOLD are spoken by HGs, and two of them, Hup and Gurindji, also appear in our sample.

### Categorization of demographics

Groups were classified as ‘HG’ if more than 50% of their food is (or was traditionally) typically obtained from hunting, gathering, and/or fishing. It is recognized that groups often exploit several strategies [48], and that for some groups the relative dependence on these strategies has fluctuated over time. In the SAM sample, contemporary cultural emphasis on hunting/gathering as opposed to farming (and fishing) was also taken into account in coding, particularly in the absence of information about past subsistence patterns. Languages where a majority of speakers live in urban environments (in the WOLD sample only) were coded distinctly.

Groups in the sample show a range of degrees of sedentism, population size, and population density. Since colonial and post-colonial impacts on population numbers make it impossible to determine precise population sizes for the case study areas, the languages were coded as ‘small’ (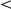
100), ‘medium’ (100–1000) or ‘large’ (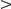
1000). Very few of these languages are likely to have had more than 5,000 speakers in pre-colonial times. Languages were also given a population density estimation of ‘low’ (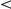
1 person per sq mile), ‘medium’ (1–25 persons per sq mile), or ‘dense’ (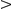
25 persons per sq mile), and were coded for whether their populations were ‘mobile’, ‘sedentary’, or ‘mixed’ (e.g. practiced seasonal mobility). For food production strategies, languages were coded for whether they obtained a majority of food by hunting and gathering or via agriculture. (See further Text S1 for details, based on Murdock [49]). These measures have been previously considered important in language change [9]. Note that due to small sample numbers it was not possible to investigate interactions in demographic factors statistically.

### Choice of data

A list of 204 items of basic vocabulary was used (see Table S3). The list was based on that used for Austronesian phylogenetics [50] with some substitutions (see details in Table S3) to maximize relevance to the case study areas. They are presumed to be culture-neutral and refer to concepts and objects that are found all over the world. Basic vocabulary is known to be maximally resistant to replacement by borrowing across languages generally [51]. Substitution of items was heavily minimized and confined only to cases where there were equivalent but slightly distinct referents in the case study regions (e.g. ‘dingo’ (*Canis dingo*) in AUS but ‘wolf’ (*Canis lupus*) in NAM). The WOLD list contains over 1500 items, and the meanings used in the area samples were extracted for comparison. The WOLD statistics thus refer to a subset of the published WOLD list.

There is some overlap (164 out of 204 items) between the 204-item list used here and the Swadesh list of basic vocabulary [52]. The list used in this study excludes concepts from the Swadesh list that are absent from the case study areas (e.g. ‘snow’), and items which are ambiguous in one or more of the case study areas (e.g. ‘we’; many of the languages in our sample have both a dual/plural distinction and an inclusive/exclusive distinction, so four words for the single word in English).

### Identification of Loans and Reconstruction Methods

Each language in the sample was coded with the aim of establishing several facts. First was the proportion of basic lexicon to have been borrowed. Languages were additionally coded for etymological sources, in order to build a profile of basic vocabulary sources. Untraceable replacement items may be unidentified loans, but they may also have other sources.

Inherited vocabulary in the language samples was reconstructed using the linguistic comparative method [44] (except in the case of linguistic isolates, where the method is not applicable). The comparative method relies on the identification of systematic correspondences between words in related languages. Sound change in language is regular; thus exceptions to regular correspondences are indicative or loans or internal analogical remodeling. For example, word-initial *f* in English regularly corresponds to word-initial *p* in Latin; cf. *fish* : *piscis*, *father* : *pater*, etc. Thus English *patron* (: Lat. *patronus*) is likely to be a loan, because it does not show the expected correspondence.

Loans between unrelated languages were identified using all appropriate methods [44], [53]. While unrelated languages may show chance resemblances in vocabulary, these are few; therefore if a word is similar in meaning and sound between two unrelated languages, it is probably a loan. The chance of loanhood is greatly increased if the word is reconstructible in one family but not in another. Loans may also be identified by their internal structure; for example, if a word is morphologically complex in one language but not in another, that is good evidence for the direction of the loan. Detection of loans proceeds in this method on a word-by-word basis and requires specialist knowledge of each region's languages and the contact history of their speakers.

The issue of potentially unidentified loans requires addressing. Loan identification methods rely on regularity of correspondences between forms (of related meaning) in related languages. It also ideally requires attestation of the word in the donor language. Thus if the donor language is not known, a loan may be undetected; further sources of undetected loans from related languages would be from words which do not show diagnostic sound changes. The latter problem was minimized by preferentially sampling from languages which border languages which are not (closely) genetically related; this makes loans easier to identify. Loans from languages which are not attested in the area are unrecoverable by definition; they would show up in our sample as ‘unique’ items (see below). Since there was no significant correlation between loan levels and unique vocabulary levels (r = −0.025, p = 0.722) in any given language, and since for any given word, its likelihood of being borrowed is not correlated with its likelihood of being a ‘unique’ item in the languages of the case study (r = 0.097, p = 0.168), the effect of undetected loans on this data sample is likely to be negligible. Since the presence of language isolates in the sample (where inherited and unique non-loans cannot be distinguished) could obscure correlations between loans and unique items, calculations were repeated with the isolates in the sample excluded. Correlations remained non-significant.

### Language Coding

Words were coded as follows: **Inheritance**. The form was inherited from an earlier stage of the language with the same meaning. **Loan** [and source] or **doubtful loan** (for example, if the word was likely to be a loan from language internal evidence, but the source could not be identified). Words which could be identified as loans in one or other of a pair of languages, but where the direction of loan was unknown, were coded but were not included in the figures analyzed here. (Figures were also calculated with all potential loans included; this did not alter any results with the exception of exogamy, which with all potential loans included was no longer statistically significant overall (p = 0.155) or in the Australian sample (p = 0.614).)

Where a loan is reconstructible as having entered the language at a period in its history prior to its split from its sister languages, it was coded as a **loan into proto-language**. This allows for the creation of a loan threshold, to minimize distortion of the sample from languages with long reconstructible histories. To count as a ‘loan’ for this dataset, the loan has to appear after the breakup of the language in question from its nearest neighbor. Thus Bardi *nimarla* ‘hand’ is reconstructible as a loan into proto-Nyulnyulan, and thus not counted as a loan into Bardi, because it is attested in other Nyulnyulan languages and has been in the family long enough to have undergone regular sound changes. **Calques** (or ‘semantic loans’) were virtually unattested in this dataset, so were not included in the loan count. In cases of **semantic shift**, the word is inherited and reconstructible within the family, but in a different meaning (e.g. Ngumbarl *nimirdi* ‘ankle’ is reconstructible to Proto-Nyulnyulan in the meaning ‘knee’). A word coded as a **unique** is not found in other regional languages and has no identifiable internal source. The unique category thus contains unidentified loans and words replaced through other word formation processes not otherwise discussed here, including *ad hoc* coinages.

**Missing** items were also noted. Most of the missing items were due to imperfect primary data. A few items had substantial missing data (‘roof’, ‘winnow/yandy’, ‘grindstone’, ‘digging stick’, ‘thick’). 16.5% of SAM case study forms are missing, while 12% are missing for AUS and 9.5% for NAM. In the SAM study, the missing forms are concentrated in a few languages; removing those languages does not affect the overall results.

In some cases, particularly in the SAM region, it was not possible to reconstruct a full history. In the case of language isolates, for example, loans can be identified with some degree of probability, but because there are no extant related languages, the comparative method cannot be used. Loans can still be identified, however, since they appear as words which are phonologically similar or identical among unrelated languages.

Data in the WOLD materials was coded only for loans, on a five-point scale of loan likelihood. For our comparison, only items considered as ‘definitely borrowed’ were included here (there were no relevant words coded as ‘probably borrowed’).

### Statistical Analyses

We used a Monte Carlo simulation approach to test the statistical significance of the differences in loan percentages for geographic and demographic groupings of the data. The simulations consisted of 1) calculating the “observed” difference in loan percentage between two groups (e.g., SAM vs. AUS), 2) pooling the loan percentages for the two groups, 3) forming two new groups of equal size to the observed groups by randomly sampling the pool with replacement, 4) calculating the difference in loan percentages between the simulated groups. P-values represent the proportion of 10,000 simulations in which the simulated difference in loan proportions exceeded the observed.

## Supporting Information

Figure S1 **Map of case study areas.**
(DOC)

Figure S2 **Summary of descriptive statistics.**
(DOC)

Table S1 **Language names, classifications, and sources.**
(DOC)

Table S2 **Language data: Etymological counts.**
(DOC)

Table S3 **Basic vocabulary list.**
(DOC)

Text S1 **Ethnographic atlas codes.**
(DOC)
