# Supplementary figures and images for: Does Lateral Transmission Obscure Inheritance in Hunter-Gatherer Languages?
Source: PLoS One. 2011 Sep 27;6(9):e25195. doi: 10.1371/journal.pone.0025195 (PMC3181316; doi:10.1371/journal.pone.0025195)

**Figure S1: Map of case study areas**


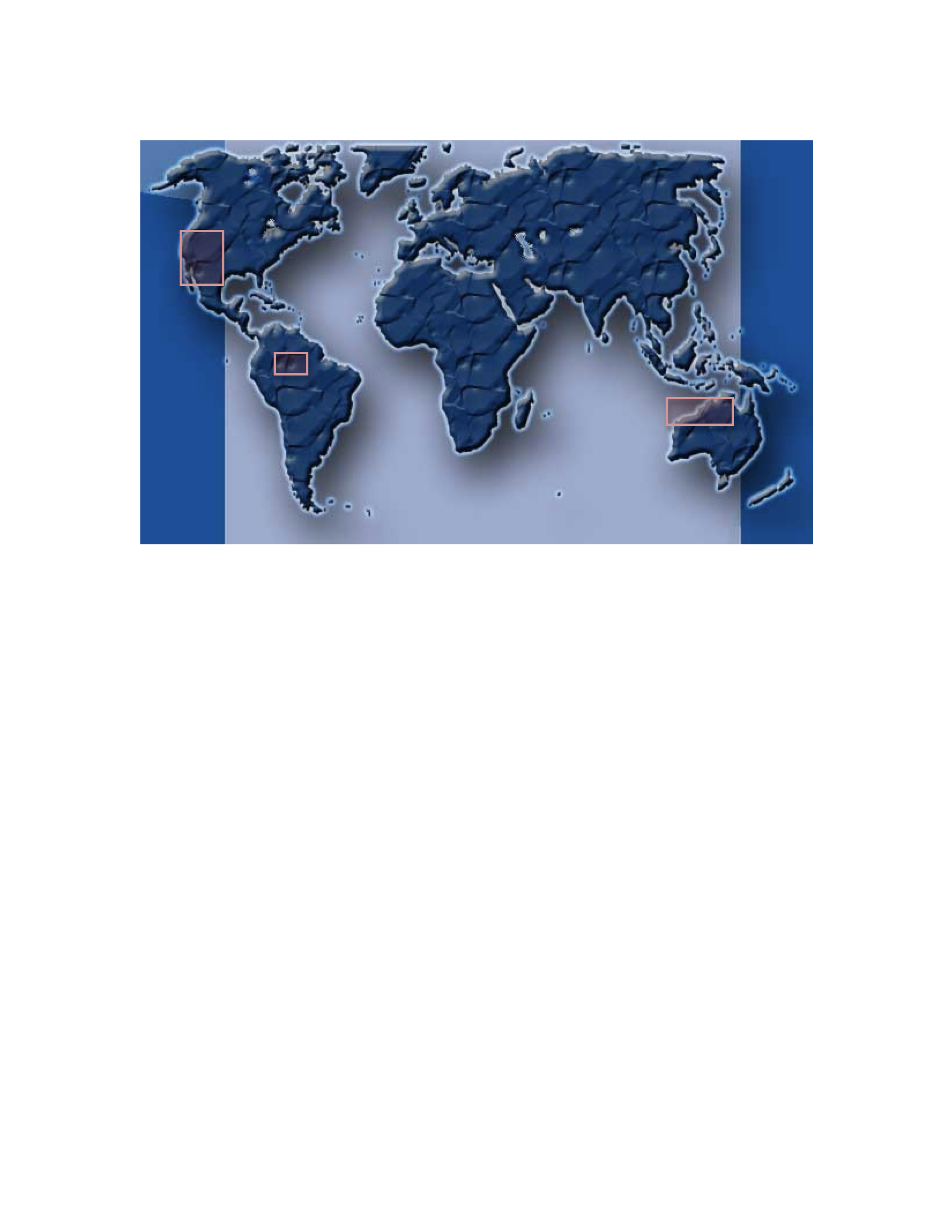

Supplement: Figure S1 — Map of case study areas. (DOC) [file pone.0025195.s001.doc]

**Figure S2 HG vs AG split by Mobility, Density, and Population size**

**
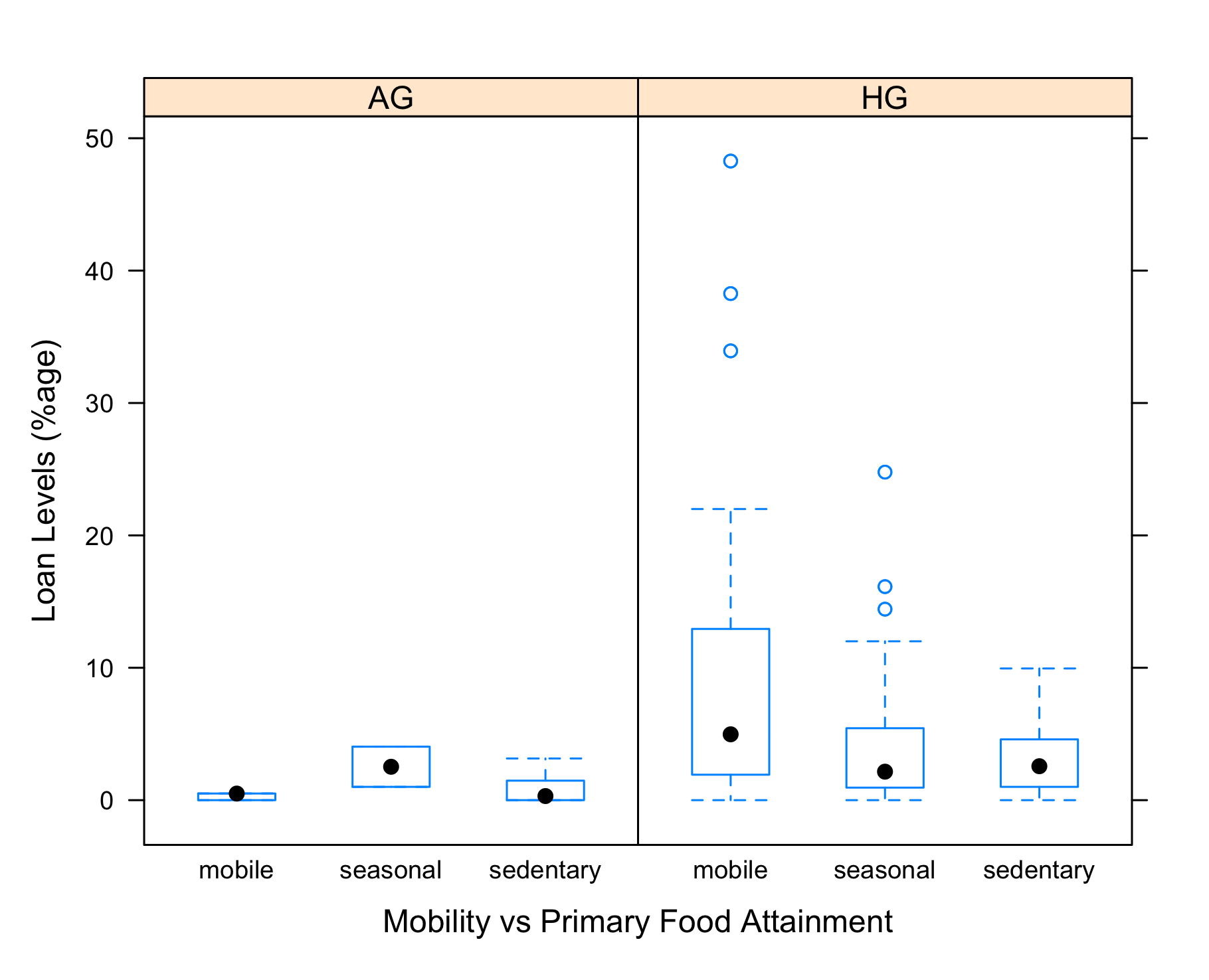
**

**
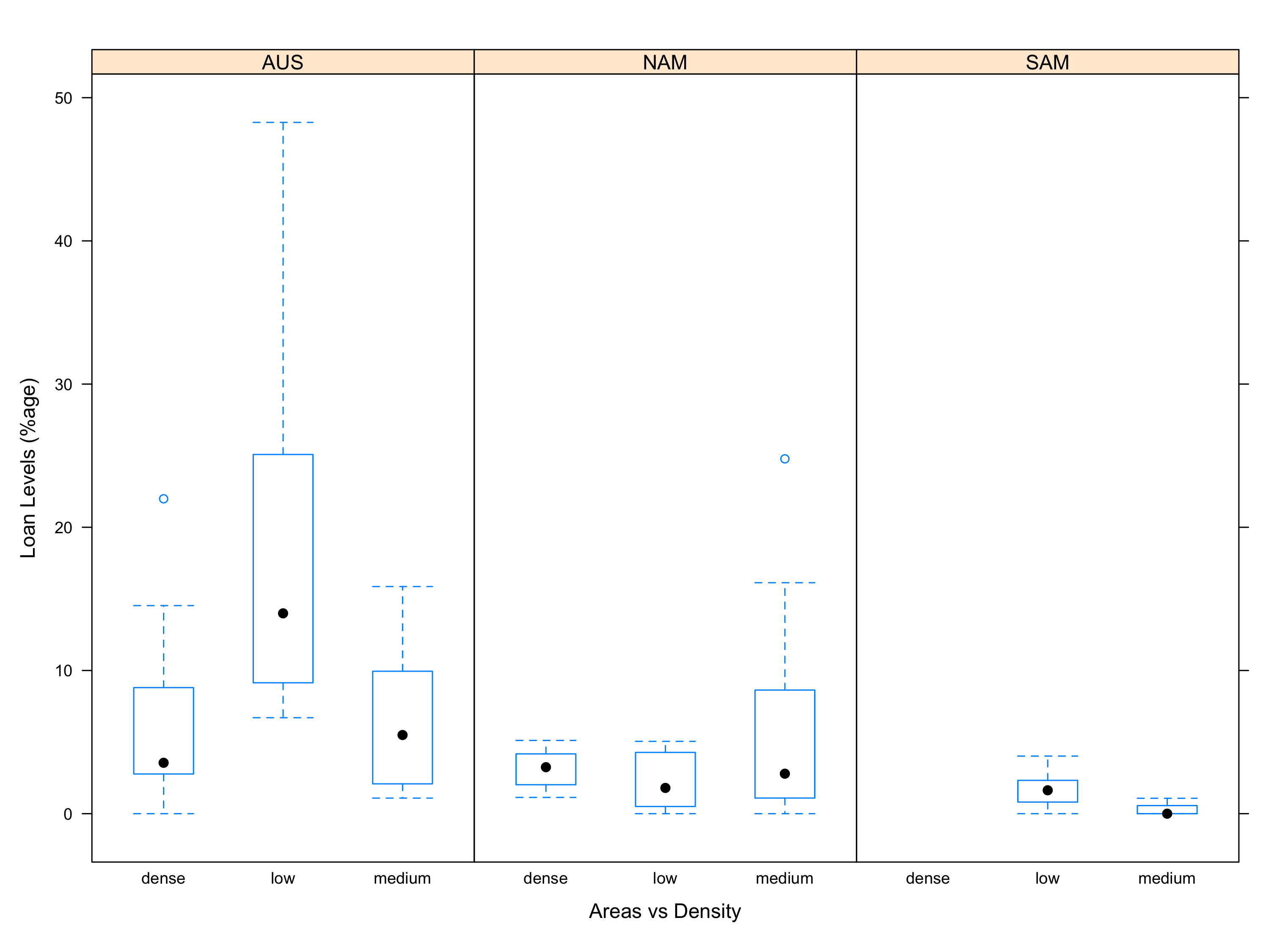
**

**
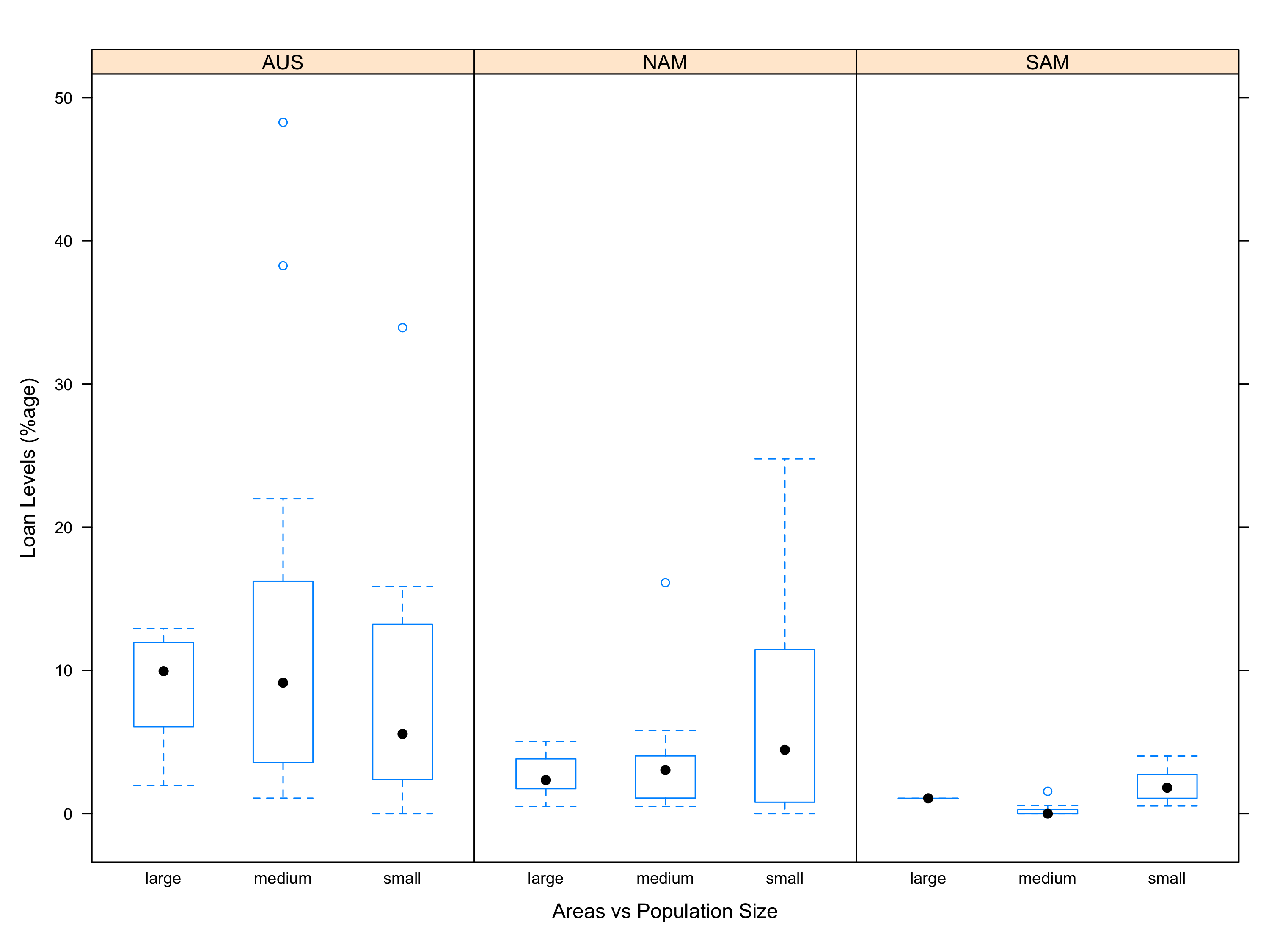
**

Supplement: Figure S2 — Summary of descriptive statistics. (DOC) [file pone.0025195.s002.doc]
