# Supplementary material for: Does Lateral Transmission Obscure Inheritance in Hunter-Gatherer Languages?
Source: PLoS One. 2011 Sep 27;6(9):e25195. doi: 10.1371/journal.pone.0025195 (PMC3181316; doi:10.1371/journal.pone.0025195)
Supplement: Table S1 — Language names, classifications, and sources. (DOC) [file pone.0025195.s003.doc]

**Table S1. Language sources**

**North Australia (AUS):**

| **Family** | **Subgroup** | **Language** | **Sources** |
| --- | --- | --- | --- |
| Nyulnyulan | Western | Bardi | Aklif 1999; Bowern 2003, 2004a |
|  |  | Nyulnyul | McGregor 1988; Nekes & Worms 1953/2006 |
|  |  | Nimanburru | Peile 1966-1967 |
|  | Eastern | Ngumbarl | Bates 1904 |
|  |  | Yawuru | Appleby et al. 1998; Hosokawa 1988, 1994 |
|  |  | Nyikina | Stokes 1982; Stokes, Johnston & Marshall 1992 |
|  |  | Warrwa | McGregor 1994-1995; Nekes & Worms 1953/2006 |
| Bunuban |  | Bunuba | Rumsey 2000 |
|  |  | Gooniyandi | McGregor 1990 |
| Jaragan |  | Gajirrabeng | Mirima Language Centre |
|  |  | Miriwoong | Kofod 1978 |
|  |  | Kija | Blythe 2001 |
| Pama-Nyungan | Marrngu | Mangala | Hudson 1973; Vaszolyi 1972 |
|  |  | Northern Mangarla | O'Grady 1967 |
|  |  | Northern Nyangumarta | McKelson 1989b |
|  |  | Nyangumarta | Nekes & Worms 1953/2006; O'Grady 1964; Sharp 2004 |
|  |  | Karajarri | McKelson 1989a; Nekes & Worms 1953/2006 |
|  | Ngumpin-Yapa | Walmajarri | Richards & Hudson 1990, Hudson 1978 |
|  |  | Mudburra | Breen 1991 |
|  |  | Gurindji | McConvell fieldnotes |
|  |  | Jaru | Tsunoda 1981, Kimberley Language Resource Centre 1992 |
|  | Yolŋu | Djapu | Morphy 1983 |
|  |  | Djinaŋ | Waters 1980, 1983, 1989 |
|  |  | Dhaŋu | Schebeck 2001; Zorc 1986 |
|  |  | Dhuwala (Gupapuyŋu) | Lowe 1960/1996; Zorc 1986 |
|  |  | Yan-nhaŋu | Bowern fieldnotes |
|  |  | Ritharrŋu | Heath 1981a |
|  | Karnic | Yandruwandha | Breen 2004 |
|  |  | Mount Freeling Diyari | Gason 1886 |
|  |  | Arabana | Hercus 1994 |
|  |  | Diyari | Austin 1981 |
|  |  | Pitta-Pitta | Blake & Breen 1971; Blake 1979 |
|  |  | Wangkayutyuru | Blake & Breen 1971; Blake 1979 |
|  |  | DiyariREU | Reuther 1973 |
|  |  | Mithaka | Breen 1971, fieldnotes |
|  |  | Karuwali | Breen 1971 |
|  |  | Ngamini | Breen 1971 |
|  |  | Yarluyandi | Breen 1971 |
|  |  | Yawarrawarrka | Reuther 1973 |
|  |  | Nhirrpi | Bowern n.d.; Breen 2004 |
|  | Karnic Fringe | Guwa | Breen 1971, 1990 |
|  |  | Yanda | Breen 1971, 1990 |
|  | Yardli | Malyangapa | Hercus & Austin 2004 |
|  |  | Wadikali | Hercus & Austin 2004 |
|  |  | Yardliyawarra | Hercus & Austin 2004 |
| Maningrida |  | Burarra | Glasgow 1994; Green 1987 |
| Gunwinygu |  | Rembarrnga | McKay n.d. |
|  |  | Ngandi | Heath 1981 |
|  |  | Wubuy (Nunggubuyu) | Heath 1980b, 1982, 1984 |

In addition, we consulted historical work from Bowern (2004b), Harvey (2009), McConvell & Laughren (2004), and McGregor & Rumsey (2009).

Aklif, Gedda. 1999. *Ardiyooloon Bardi ngaanka: One Arm Point Bardi dictionary*. Halls Creek, Western Australia: Kimberley Language Resource Centre.

Appleby, Dianne, Doris Edgar, Elsie Edgar, Susan Edgar & Thelma Saddler. 1998. *Yawuru Ngan-ga: A phrasebook of the Yawuru Language*. Broome, Australia: Magabala Books.

Austin, Peter. 1981. *A grammar of Diyari, South Australia*. Cambridge: Cambridge University Press.

Bates, Daisy. 1904. Native vocabularies: Broome magisterial district. Canberra: National Library of Australia, ms.

Blake, Barry J. 1979. Pitta-Pitta. In R. M. W. Dixon & Barry J. Blake (eds.), *Handbook of Australian languages*, vol. 1, 182-242. Canberra: Australian National University Press.

Blake, Barry J. & Gavan Breen. 1971. *The Pitta-Pitta dialects*. (Linguistic Communications 4). Melbourne: Monash University.

Blythe, Joseph. 2001. *Yuwurriyangem Kijam (Our language Kija): A phrasebook of the Kija language*. Halls Creek, Western Australia: Kimberley Language Resource Centre.

Bowern, Claire. n.d. Nhirrpi sketch grammar. ms.

Bowern, Claire. 2003. Supplement to Ardiyooloon Bardi ngaanka: One Arm Point Bardi dictionary. ms.

Bowern, Claire. 2004a. *Bardi verb morphology in historical perspective.* Cambridge, MA: Harvard University PhD dissertation.

Bowern, Claire. 2004b. Diagnostic similarities and differences between Nyulnyulan and neighbouring languages. In Claire Bowern & Harold Koch (eds.), *Australian languages: Classification and the comparative method*, 295-318. (Current Issues in Linguistic Theory 249). Amsterdam: John Benjamins.

Breen, Gavan. 1971. *Aboriginal languages of western Queensland (preliminary version)*. (Linguistic Communications 5). Melbourne: Monash University.

Breen, Gavan. 1990. *Salvage studies of a number of extinct Aboriginal languages of western Queensland*. (Pacific Linguistics B-105). Canberra: Pacific Linguistics.

Breen, Gavan. 1991. Mudburra graded wordlist. Aboriginal Studies Electronic Data Archive (ASEDA) 0269, ms.

Breen, Gavan. 2004. *Innamincka words: Yandruwandha dictionary and stories*. (Pacific Linguistics 559). Canberra: Pacific Linguistics.

Gason, Samuel. 1886. From Mount Freeling to Pirigundi Lake. In Edward M. Curr (ed.), *The Australian race: Its origin, languages, customs, place of landing in Australia, and the routes by which it spread itself over that continent*, vol. 2, 44-107. Melbourne: John Ferres.

Glasgow, Kathleen. 1994. *Burarra-Gun-nartpa dictionary: With English finder list*. Darwin, Australia: Summer Institute of Linguistics, Australian Aborigines and Islanders Branch.

Green, Rebecca. 1987. *A sketch grammar of Burarra.* Canberra: Australian National University BA honors thesis.

Harvey, Mark. 2009. The genetic status of Garrwan. *Australian Journal of Linguistics* 29(2). 195-244.

Heath, Jeffrey. 1980a. *Basic materials in Ritharngu: Grammar, texts, and dictionary*. (Pacific Linguistics B-62). Canberra: Pacific Linguistics.

Heath, Jeffrey. 1980b. *Nunggubuyu myths and ethnographic tests*. (AIAS New Series 23). Canberra: Australian Institute of Aboriginal Studies.

Heath, Jeffrey. 1981. A case of intensive lexical diffusion: Arnhem Land, Australia. *Language* 57(2). 335-367.

Heath, Jeffrey. 1982. *Nunggubuyu dictionary*. (AIAS New Series 36). Canberra: Australian Institute of Aboriginal Studies.

Heath, Jeffrey. 1984. *Functional grammar of Nunggubuyu*. Canberra: Australian Institute of Aboriginal Studies.

Hercus, Luise. 1994. *A grammar of the Arabana-Wangkangurru language, Lake Eyre Basin, South Australia*. (Pacific Linguistics C-128). Canberra: Pacific Linguistics.

Hercus, Luise & Peter Austin. 2004. The Yarli languages. In Claire Bowern & Harold Koch (eds.), *Australian languages: Classification and the comparative method*, 179-206. (Current Issues in Linguistic Theory 249). Amsterdam: John Benjamins.

Hosokawa, Komei. 1988. Classified Yawuru dictionary. Australian Institute of Aboriginal and Torres Islander Studies MS 2605, ms.

Hosokawa, Komei. 1994. Meanings in Yawuru: A semantically oriented description of an indigenous language of Kimberley, Western Australia. Aboriginal Studies Electronic Data Archive (ASEDA) 0753. Revised version of the author’s 1991 ANU PhD dissertation, ms.

Hudson, Joyce. 1973. *Mangarla word list*. Darwin, Australia: Summer Institute of Linguistics, Australian Aborigines Branch.

Hudson, Joyce. 1978. *The core of Walmatjari grammar*. Canberra: Australian Institute of Aboriginal Studies.

Kimberley Language Resource Centre. 1992. *Jaru dictionary*. Draft ed. Halls Creek, Western Australia: Kimberley Language Resource Centre.

Kofod, F. M. 1978. *The Miriwung language (East Kimberley): A phonological and morphological study.* Armidale, Australia: University of New England MA thesis.

Lowe, Beulah. 1960/1996. *Grammar lessons in Gupapuyngu*. Darwin: Aboriginal Resource & Development Services.

McConvell, Patrick & Mary Laughren. 2004. The Ngumpin-Yapa subgroup. In Claire Bowern & Harold Koch (eds.), *Australian languages: Classification and the comparative method*, 151-177. (Current Issues in Linguistic Theory 249). Amsterdam: John Benjamins.

McGregor, William. 1988. Field notebooks in Nyulnyul and Unggumi, 1988. Australian Institute of Aboriginal and Torres Islander Studies MS 2879.

McGregor, William. 1990. *A functional grammar of Gooniyandi*. (Studies in Language Companion Series 22). Amsterdam: John Benjamins.

McGregor, William. 1994-1995. Warrwa notebooks. Australian Institute of Aboriginal and Torres Islander Studies MS 3348.

McGregor, William & Alan Rumsey. 2009. *Worrorran revisited: The case for genetic relations among languages of the Northern Kimberley region of Western Australia*. (Pacific Linguistics 600). Canberra: Pacific Linguistics.

McKay, Graham. n.d. Rembarrnga dictionary and grammar. Aboriginal Studies Electronic Data Archive (ASEDA) 0607, ms.

McKelson, Kevin R. 1989a. Topical vocabulary of Northern Nyangumarta. Aboriginal Studies Electronic Data Archive (ASEDA) 0017, ms.

McKelson, Kevin R. 1989b. Karajarri wordlist. Aboriginal Studies Electronic Data Archive (ASEDA) 0069, ms.

Morphy, Frances. 1983. Djapu, a Yolngu dialect. In R. M. W. Dixon & Barry J. Blake (eds.), *Handbook of Australian languages*, vol. 3, 1-188. Canberra: Australian National University Press.

Nekes, Hermann & Ernest A. Worms. 1953/2006. *Australian languages*. (Ed.) William B. McGregor. Berlin: Mouton de Gruyter.

O’Grady, Geoffrey N. 1964. *Nyangumata grammar*. (Oceania Linguistic Monographs 9). Sydney: University of Sydney.

O’Grady, Geoffrey N. 1967. Northern Mangarla wordlist. Aboriginal Studies Electronic Data Archive (ASEDA) 0192, ms.

Peile, Anthony Rex. 1966-1967. *Language elicitation*. Audio recording. Australian Institute of Aboriginal and Torres Islander Studies PEILE_A05 - 00454A.

Reuther, J. G. 1973. *Three central Australian grammars: Diari, Jandruwanta, Wonkanguru (part of volume V of the Reuther manuscript in the South Australian Museum)*. (Ed.) L. A. Hercus & J. G. Breen. (Trans.) T. Schwarzschild & L. A. Hercus. Canberra: Australian Institute of Aboriginal Studies.

Richards, Eirlys & Joyce Hudson. 1990. *Walmajarri – English dictionary with English finder list.* Darwin, Australia: Summer Institute of Linguistics.

Rumsey, Alan. 2000. Bunuba. In R. M. W. Dixon & Barry J. Blake (eds.), *The handbook of Australian languages*, vol. 5, 34-153. Oxford: Oxford University Press.

Schebeck, Bernhard. 2001. *Dialect and social groupings in northeast Arnheim [i.e. Arnhem] Land*. (Ed.) R. M. W. Dixon. (LINCOM Studies in Australian Languages 7). Muenchen: LINCOM Europa.

Sharp, Janet. 2004. *Nyangumarta: A language of the Pilbara region of Western Australia*. (Pacific Linguistics 556). Canberra: Pacific Linguistics.

Stokes, Bronwyn. 1982. *A description of Nyigina: A language of the West Kimberley, Western Australia.* Canberra: Australian National University PhD dissertation.

Stokes, Bronwyn, Gladys Johnston & Lucy Marshall. 1992. Nyikina-English: A first lexicon. Aboriginal Studies Electronic Data Archive (ASEDA) 0472, ms.

Tsunoda, Tasaku. 1981. *The Djaru language of Kimberley, Western Australia*. (Pacific Lingusitics B-78). Canberra: Pacific Linguistics.

Vaszolyi, Eric G. 1972. Mangala vocabulary. Australian Institute of Aboriginal and Torres Islander Studies MS 426.

Waters, Bruce E. 1980. Djinang phonology. In *Papers in Australian linguistics no. 14*, 1-71. (Pacific Linguistics A-60). Canberra: Pacific Linguistics.

Waters, Bruce E. 1983. *An interim Djinang dictionary*. (Work Papers of SIL-AAB, Series B 9). Darwin, Australia: Summer Institute of Linguistics.

Waters, Bruce E. 1989. *Djinang and Djinba, a grammatical and historical perspective* (Pacific Linguistics C-114). Canberra: Pacific Linguistics.

Zorc, R. David. 1986. *Yolngu-Matha dictionary*. Batchelor, Australia: School of Australian Linguistics, Darwin Institute of Technology.

**California and the Great Basin (NAM):**

| **Family** | **Subgroup** | **Language** | **Sources** |
| --- | --- | --- | --- |
| Chumash |  | Chumash Barbareño | Applegate 1972, 1976; Beeler 1967, 1976, 1978; Harrington 1974, fieldnotes; Klar 1977, 1980, 1981; Mithun 1998; Ono 1996; Wash 2001 |
|  |  | Chumash Cruzeño |  |
|  |  | Chumash Ineseño |  |
|  |  | Chumash Obispeño |  |
|  |  | Chumash Ventureño |  |
| Hokan |  | Esselen | Heizer 1952; Kroeber 1904; Shaul 1988, 1995; Turner & Shaul 1982 |
|  |  | Washo | Jacobsen 1958, 1964, 1979; Kroeber 1907a; Washo Project 2008 |
|  |  | Salinan | Mason 1918; Turner 1980, 1983 |
| Isolate |  | Seri Comcaac | Moser & Marlett 2005 |
| Penutian | Yok-Utian | Yokuts Yowlumne | Berman 2002; Gamble 1991; Golla 1964; Kroeber 1907b, 1963; Newman 1944, 1964; Whistler & Golla 1986 |
|  |  | Yokuts Palewyami |  |
|  |  | Yokuts Yawdanchi |  |
|  |  | Central Sierra Miwok | Broadbent 1964; Callaghan 1962, 1964, 1965, 1967, 1970, 1972, 1984, 1987, 1997, 2001; Freeland & Broadbent 1960 |
|  |  | Northern Sierra Miwok |  |
|  |  | Southern Sierra Miwok |  |
|  |  | Plains Miwok |  |
|  |  | Lake Miwok |  |
|  |  | Mutsun | Callaghan 1962, 1967, 1988, 1992, 1997, 2001; Okrand 1977; Warner n.d. |
|  | Maiduan | Maidu | Shipley 1961, 1963; Uldall & Shipley 1966; Ultan 1964 |
|  |  | Nisenan |  |
|  | Wintun | Wintu | Golla 1977; Pitkin 1985; Schlichter 1981; Shepherd 2006 |
| Uto-Aztecan (Northern branch) | Numic | Comanche | Bethel et al. 1993; Dayley 1989; Fowler 1972; Harrington n.d.a; Nichols 1974; Press 1979; Snapp, Anderson & Anderson 1982; Thornes 2003; Zigmond, Booth & Munro 1991 |
|  |  | Big Smokey Valley Shoshone |  |
|  |  | Tümpisa Shoshone |  |
|  |  | Kawaiisu |  |
|  |  | Southern Paiute |  |
|  |  | Southern Ute |  |
|  |  | Chemehuevi |  |
|  |  | Western Mono |  |
|  |  | Northern Paiute |  |
|  | Kern | Bankalachi Toloim | Hill 2009; Kroeber 1907c; Merriam 1932-1935; C. Voegelin 1935; E. Voegelin 1938 |
|  |  | Tübatulabal |  |
|  | Takic | Cahuilla | Anderton 1988; Bright 1968; Elliott 1999; Harrington n.d.b; J. Hill 2005; J. Hill and Nolasquez 1972; K. Hill 2001, 2003; Mamet 2008; McCawley 1996; Seiler 1977; Seiler and Hioki 1979 |
|  |  | Cupeño |  |
|  |  | Luiseño |  |
|  |  | Serrano |  |
|  |  | Gabrielino |  |
|  |  | Kitanemuk |  |
| Yukian |  | Wappo | Elmendorf 1968, 1997; Elmendorf & Shepherd 1999; Oswalt 1979; Radin 1929; Sawyer 1965, 1991; Schlichter 1985; Shipley 1957; Thompson, Park & Li 2006 |
|  |  | Yuki |  |
| Yuman |  | Cocopa | Couro and Hutcheson 1973; Crawford 1989; Gordon 1986; Halpern 1946a,b,c, 1947a,b; Langdon 1970, 1976a,b, 1977, 1979, 1985; Langdon & Munro 1980; Mixco 1978, 1979, 1985; Munro, Brown & Crawford 1992; Shaterian 1983 |
|  |  | ʼIipay Aa |  |
|  |  | Kiliwa |  |
|  |  | Mojave |  |
|  |  | Yavapai |  |
|  |  | Yuma |  |

Sources on Pomoan (Grekoff 1964; McLendon 1964; Moshinsky 1970; Oswalt 1964, 1976; Vihmann 1970) were consulted in order to identify loanwords from the group into Yukian, Utian, and Wintun languages. However, Pomoan languages were not included in the sample. Three languages of Northwest Mexico (Cocopa and Kiliwa in the Yuman family, and Seri, an isolate) were included for comparative purpose. Yavapai, in Arizona, was also included in order to round out the Yuman sample. Yuma, Mojave, Yavapai, and Cocopa (Yuman) as well as Chemehuevi (Uto-Aztecan) in the southeast are spoken in communities that practiced horticulture.

Both comparativist and arealist studies have a 100-year-long history in the area. Among the most significant comparativist sources are Miller 1988 and Stubbs 2008 for Uto-Aztecan; Callaghan 1962, 1964, 1967, 1972, 1997, 2001; Golla 1964; and Whistler & Golla 1986 for Yok-Utian; Langdon 1970, 1976a,b , 1977, 1979, 1985 and Mixco 1978, 1979 for Yuman; and Shepherd 2006 for Wintun. Several languages in the sample are not documented in dictionaries; where this was the case, lexical material was retrieved from grammatical studies and from archived field notes, especially those of J. P. Harrington for the Chumashan languages and C. H. Merriam for Bankalachi/Toloim, one of the Kern (Uto-Aztecan) languages.

Anderton, Alice Jeanne. 1988. The language of the Kitanemuks of California. Los Angeles: University of California PhD dissertation.

Applegate, Richard B. 1972. Ineseño Chumash grammar. Berkeley, CA: University of California PhD dissertation.

Applegate, Richard B. 1976. Reduplication in Chumash. In Margaret Langdon & Shirley Silver (eds.), *Hokan Studies: Papers from the First Conference on Hokan Languages held in San Diego, California, April 23-25, 1970*, 271-284. (Janua Linguarum: Series Practica 181). The Hague: Mouton.

Beeler, Madison S. 1976. Barbareño Chumash grammar: A farrago. In Margaret Langdon & Shirley Silver (eds.), *Hokan Studies: Papers from the First Conference on Hokan Languages held in San Diego, California, April 23-25, 1970*, 251-270. (Janua Linguarum: Series Practica 181). The Hague: Mouton.

Beeler, Madison S. 1978. Barbareño Chumash text and lexicon. In Mohammad Ali Jazayery, Edgar C. Polomé & Werner Winter (eds.), *Linguistic and literary studies in honor of Archibald A. Hill, vol. 2: Descriptive linguistics*, 171-194. The Hague: Mouton.

Beeler, Madison S. (ed.). 1967. *The Ventureño confesionario of José Señán, O.F.M.* (University of California Publications in Linguistics 47). Berkeley, CA: University of California Press.

Berman, Howard. 2002. Merriam’s Palewyami vocabulary. *International Journal of American Linguistics* 68(4). 428-446.

Bethel, Rosalie, Paul V. Kroskrity, Christopher Loether & Gregory Reinhardt. 1993. A dictionary of Western Mono, 2nd edn. Pocatello, ID: Idaho State University, ms.

Bright, William. 1964. *Studies in Californian linguistics*. (University of California Publications in Linguistics 34). Berkeley, CA: University of California Press.

Bright, William. 1968. *A Luiseño dictionary*. (University of California Publications in Linguistics 51). Berkeley, CA: University of California Press.

Broadbent, Sylvia M. 1964. *The Southern Sierra Miwok language*. (University of California Publications in Linguistics 38). Berkeley, CA: University of California Press.

Callaghan, Catherine A. 1962. Comparative Miwok-Mutsun with notes on Rumsen. *International Journal of American Linguistics* 28(2). 97-107.

Callaghan, Catherine A. 1964. Phonemic borrowing in Lake Miwok. In William Bright (ed.), *Studies in Californian linguistics*, 46-53. (University of California Publications in Linguistics 34). Berkeley, CA: University of California Press.

Callaghan, Catherine A. 1965. *Lake Miwok Dictionary*. (University of California Publications in Linguistics 39). Berkeley, CA: University of California Press.

Callaghan, Catherine A. 1967. Miwok-Costanoan as a subfamily of Penutian. *International Journal of American Linguistics* 33(3). 224-227.

Callaghan, Catherine A. 1970. *Bodega Miwok dictionary*. (University of California Publications in Linguistics 60). Berkeley, CA: University of California Press.

Callaghan, Catherine A. 1972. Proto-Miwok phonology. *General Linguistics* 12. 1-31.

Callaghan, Catherine A. 1984. *Plains Miwok dictionary*. (University of California Publications in Linguistics 105). Berkeley, CA: University of California Press.

Callaghan, Catherine A. 1987. *Northern Sierra Miwok dictionary*. (University of California Publications in Linguistics 110). Berkeley, CA: University of California Press.

Callaghan, Catherine A. 1988. Karkin revisited. *International Journal of American Linguistics* 54(4). 436-452.

Callaghan, Catherine A. 1992. The riddle of Rumsen. *International Journal of American Linguistics* 58(1). 36-48.

Callaghan, Catherine A. 1997. Evidence for Yok-Utian. *International Journal of American Linguistics* 63(1). 18-64.

Callaghan, Catherine A. 2001. More Evidence for Yok-Utian: A Reanalysis of the Dixon and Kroeber Sets. *International Journal of American Linguistics* 67(3). 313-345.

Conathan, Lisa Jane. 2004. The linguistic ecology of northwestern California: Contact, functional convergence and dialectology. Berkeley, CA: University of California PhD dissertation.

Couro, Ted & Christina Hutcheson. 1973. *Dictionary of Mesa Grande Diegueño: ’Iipay Aa-English/English-’Iipay Aa*. Banning, CA: Malki Museum Press.

Crawford, James M. 1989. *Cocopa dictionary*. (University of California Publications in Linguistics 114). Berkeley, CA: University of California Press.

Crawford, Judith G. 1976. Seri and Yuman. In Margaret Langdon & Shirley Silver (eds.), *Hokan Studies: Papers from the First Conference on Hokan Languages held in San Diego, California, April 23-25, 1970*, 305-324. (Janua Linguarum: Series Practica 181). The Hague: Mouton.

Dayley, Jon P. 1989. *Tümpisa (Panamint) Shoshone grammar*. (University of California Publications in Linguistics 115). Berkeley, CA: University of California Press.

Elliott, Eric. 1999. Dictionary of Rincón Luiseño. San Diego, CA: University of California PhD dissertation.

Elmendorf, William W. 1968. Lexical and cultural change in Yukian. *Anthropological Linguistics* 10(7). 1-41.

Elmendorf, William W. 1997. A preliminary analysis of Yukian root structure. *Anthropological Linguistics* 39(1). 74-91.

Elmendorf, William W. & Alice Shepherd. 1999. Another look at Wappo-Yuki loans. *Anthropological Linguistics* 41(2). 209-229.

Fowler, Catherine Louise Sweeney. 1972. Comparative Numic ethnobiology. Pittsburgh, PA: University of Pittsburgh PhD dissertation.

Freeland, L. S. & Sylvia M. Broadbent. 1960. *Central Sierra Miwok dictionary, with texts*. (University of California Publications in Linguistics 23). Berkeley, CA: University of California Press.

Gamble, Geoffrey. 1991. Palewymani: A Yokuts key. In Sandra Chung & Jorge Hankamer (eds.), *A festschrift for William F. Shipley*, 61-81. Santa Cruz, CA: Syntax Research Center.

Gamble, Lynn H. 2008. *The Chumash world at European contact: Power, trade, and feasting among complex hunter-gatherers*. Berkeley, CA: University of California Press.

Golla, Victor K. 1964. Comparative Yokuts phonology. In William Bright (ed.), *Studies in Californian linguistics*, 54-66. (University of California Publications in Linguistics 34). Berkeley, CA: University of California Press.

Golla, Victor K. 1997. The Alsea-Wintuan connection. *International Journal of American Linguistics* 63(1). 157-170.

Gordon, Lynn. 1986. *Maricopa morphology and syntax*. (University of California Publications in Linguistics 108). Berkeley, CA: University of California Press.

Grekoff, George V. 1964. A note on comparative Pomo. In William Bright (ed.), *Studies in Californian linguistics*, 67-72. (University of California Publications in Linguistics 34). Berkeley, CA: University of California Press.

Halpern, A. M. 1946a. Yuma I: Phonemics. *International Journal of American Linguistics* 12(1). 25-33.

Halpern, A. M. 1946b. Yuma II: Morphophonemics. *International Journal of American Linguistics* 12(3). 147-151.

Halpern, A. M. 1946c. Yuma III: Grammatical Processes and the Noun. *International Journal of American Linguistics* 12(4). 204-212.

Halpern, A. M. 1947a. Yuma IV: Verb Themes. *International Journal of American Linguistics* 13(1). 18-30.

Halpern, A. M. 1947b. Yuma V: Conjugation of the Verb Theme. *International Journal of American Linguistics* 13(2). 92-107.

Harrington, John P. n.d.a Chemehuevi noun list. Ditto copy in possession of Jane H. Hill, ms.

Harrington, John P. n.d.b Gabrielino field notes. In possession of Kenneth C. Hill, ms.

Harrington, John P. n.d.c Papers. National Anthropological Archives, Smithsonian Institution, Washington, DC, ms.

Harrington, John P. 1974. Sibilants in Ventureño. *International Journal of American Linguistics* 40(1). 1-9.

Heizer, R. F. 1952. The mission Indian vocabularies of Alphonse Pinart. *Anthropological Records* 15(1). (California Indian Linguistic Records 1). 1-84.

Hill, Jane H & Rosinda Nolasquez. 1973. *Muluʼwetam: The first people: Cupeño oral history and language*. Banning, CA: Malki Museum Press.

Hill, Jane H. 2005. *A grammar of Cupeño*. (University of California Publications in Linguistics 136). Berkeley, CA: University of California Press.

Hill, Kenneth C. 2001. Serrano dictionary draft. Tuscon, AZ, ms.

Hill, Kenneth C. 2003. A typological sketch of Serrano. Tuscon, AZ, ms.

Hill, Kenneth C. 2009. Tübatulabal dictionary. Tuscon, AZ, ms.

Hinton, Leanne. 1991. Takic and Yuman: A study in phonological convergence. *International Journal of American Linguistics* 57(2). 133-15

Jacobsen, William H., Jr. 1958. Washo and Karok: An approach to comparative Hokan. *International Journal of American Linguistics* 24(3). 195-212.

Jacobsen, William H., Jr. 1964. A grammar of the Washo language. Berkeley, CA: University of California PhD dissertation.

Jacobsen, William H., Jr. 1979. Hokan inter-branch comparison. In Lyle Campbell & Marianne Mithun (eds.), *The languages of Native America: Historical and comparative assessment*, 545-591. Austin, TX: University of Texas Press.

Johnson, John R. & Joseph G. Lorenz. 2006. Genetics, linguistics, and prehistoric migrations: An analysis of California Indian mitochondrial DNA lineages. *Journal of California and Great Basin Anthropology* 26(1). 33–64.

Klar, Kathryn. 1977. Topics in historical Chumash grammar. Berkeley, CA: University of California.

Klar, Kathryn. 1980. Northern Chumash numerals. In Kathryn Klar, Margaret Langdon & Shirley Silver (eds.), *American Indian and Indoeuropean studies: Papers in honor of Madison S. Beeler*, 113-119. The Hague: Mouton.

Klar, Kathryn. 1981. Proto-Chumash person and number markers. In James E. Redden (ed.), *Proceedings of the 1980 Hokan Languages Workshop held at University of California, Berkeley, June 30-July 2, 1980*, 86-95. (Occasional Papers on Linguistics 9). Carbondale, IL: Department of Linguistics, Southern Illinois University.

Kroeber, A. L. 1904. The languages of the coast of California south of San Francisco. *University of California Publications in American Archaeology and Ethnology* 2(2). 29-80.

Kroeber, A. L. 1907a. The Washo language of east central California and Nevada. *University of California Publications in American Archaeology and Ethnology* 4(5). 251-317.

Kroeber, A. L. 1907b. The Yokuts language of south central California. *University of California Publications in American Archaeology and Ethnology* 2(5). 165-393.

Kroeber, A. L. 1907c. Shoshonean dialects of California. *University of California Publications in American Archaeology and Ethnology* 4(3). 65-165.

Kroeber, A. L. 1911. The languages of the coast of California north of San Francisco. *University of California Publications in American Archaeology and Ethnology* 9(3). 273-435.

Kroeber, A. L. 1963. Yokuts dialect survey. *Anthropological Records* 11(3). 177-251.

Langdon, Margaret. 1970. Review of Alan Campbell Wares, A comparative study of Yuman consonantism. *Language* 46(2). 533-544. doi:10.2307/412302.

Langdon, Margaret. 1976a. The Proto-Yuman vowel system. In Margaret Langdon & Shirley Silver (eds.), *Hokan Studies: Papers from the First Conference on Hokan Languages held in San Diego, California, April 23-25, 1970*, 129-148. (Janua Linguarum: Series Practica 181). The Hague: Mouton.

Langdon, Margaret. 1976b. Metathesis in Yuman languages. *Language* 52(4). 866-883. doi:10.2307/413299.

Langdon, Margaret. 1977. Stress, length, and pitch in Yuman languages. In Larry M. Hyman (ed.), *Studies in stress and accent*, 239-259. (Southern California Occasional Papers in Linguistics 4). Los Angeles: Department of Linguistics, University of Southern California.

Langdon, Margaret. 1979. Some thoughts on Hokan with particular reference to Pomoan and Yuman. In Lyle Campbell & Marianne Mithun (eds.), *The languages of Native America: Historical and comparative assessment*, 592-649. Austin, TX: University of Texas Press.

Langdon, Margaret. 1985. Did Proto-Yuman have a prefix *aː-? In Ursula Pieper & Gerhard Stickel (eds.), *Studia linguistica diachronica et synchronica: Werner Winter, sexagenario anno MCMLXXXIII*, 503-517. Berlin: Mouton de Gruyter.

Langdon, Margaret & Pamela Munro. 1980. Yuman numerals. In Kathryn Klar, Margaret Langdon & Shirley Silver (eds.), *American Indian and Indoeuropean studies: Papers in honor of Madison S. Beeler*, 113-119. The Hague: Mouton.

Mamet, Ingo. 2008. *Man-bear travels to hell: Aspects of the phonological description of a Cahuilla narrative*. (Languages of the World: Text Collections 27). München: Lincom Europa.

Mason, J. Alden. 1918. The language of the Salinan Indians. *University of California Publications in American Archaeology and Ethnology* 14(1). 1-154.

McCawley, William. 1996. *The first Angelinos: The Gabrielino Indians of Los Angeles*. Banning, CA: Malki Museum Press.

McLendon, Sally. 1964. Northern Hokan (b) and (c): A comparison of Eastern Pomo and Yana. In William Bright (ed.), *Studies in Californian linguistics*, 126-144. (University of California Publications in Linguistics 34). Berkeley, CA: University of California Press.

Merriam, C. Hart. 1930. A remarkable case of word borrowing among California Indians. *Science* 71(1847). 546.

Merriam, C. Hart. 1932-1935. Vocabularies of North American Indians: Pahn-kă-lă-che. http://www.archive.org/stream/bancroft_chartmerriam_1556_49 (18 July, 2011).

Miller, Wick R. 1988. Uto-Aztecan cognate sets. Cross-checked, edited, and amended by Kenneth C. Hill through 2010. University of Utah, ms.

Mithun, Marianne. 1998. The regression of sibilant harmony through the life of Barbareño Chumash. In Jane H Hill, P. J Mistry & Lyle Campbell (eds.), *The life of language: Papers in linguistics in honor of William Bright*, 221-241. (Trends in Linguistics: Studies and Monographs 108). Berlin: Mouton de Gruyter.

Mixco, Mauricio J. 1978. *Cochimí and Proto-Yuman: Lexical and syntactic evidence for a new language family in lower California*. (University of Utah Anthropological Papers 101). Salt Lake City, UT: University of Utah Press.

Mixco, Mauricio J. 1979. Northern Cochimí dialectology and Proto-Yuman. *Journal of California and Great Basin Anthropology* 1. 39-64.

Mixco, Mauricio J. 1985. *Kiliwa dictionary*. (University of Utah Anthropological Papers 109). Salt Lake City, UT: University of Utah Press.

Moser, Mary Beck & Stephen A. Marlett. 2005. *Comcáac quih yaza quih hant ihíip hac: Cmiique iitom, cocsar iitom, maricáana iitom (Diccionario seri-español-inglés: Con índices español-seri, inglés-seri y con gramática)*. Hermosillo, Mexico: Secretaria de Educación y Cultura, Universidad de Sonora.

Moshinsky, Julius. 1976. Historical Pomo phonology. In Margaret Langdon & Shirley Silver (eds.), *Hokan Studies: Papers from the First Conference on Hokan Languages held in San Diego, California, April 23-25, 1970*, 55-76. (Janua Linguarum: Series Practica 181). The Hague: Mouton.

Munro, Pamela, Nellie Brown & Judith G. Crawford. 1992. *A Mojave dictionary*. (UCLA Occasional Papers in Linguistics 10). Los Angeles: Department of Linguistics, University of California, Los Angeles.

Newman, Stanley. 1944. *Yokuts language of California*. (Viking Fund Publications in Anthropology 2). New York: Viking Fund.

Newman, Stanley. 1964. Comparison of Zuni and California Penutian. *International Journal of American Linguistics* 30(1). 1-13.

Nichols, Michael Porter. 1974. Northern Paiute historical grammar. Berkeley, CA: University of California PhD dissertation.

O’Neill, Sean. 2008. *Cultural contact and linguistic relativity among the Indians of northwestern California*. Norman, OK: University of Oklahoma Press.

Okrand, Marc. 1977. Mutsun grammar. Berkeley, CA: University of California PhD dissertation.

Okrand, Marc. 1989. More on Karkin and Costanoan. *International Journal of American Linguistics* 55(2). 254-258.

Ono, Tsuyoshi. 1996. Information flow and grammatical structure in Barbareño Chumash. Santa Barbara, CA: University of California PhD dissertation.

Oswalt, Robert L. 1964. A comparative study of two Pomo languages. In William Bright (ed.), *Studies in Californian linguistics*, 149-162. (University of California Publications in Linguistics 34). Berkeley, CA: University of California Press.

Oswalt, Robert L. 1976. Comparative verb morphology of Pomo. In Margaret Langdon & Shirley Silver (eds.), *Hokan Studies: Papers from the First Conference on Hokan Languages held in San Diego, California, April 23-25, 1970*, 13-28. (Janua Linguarum: Series Practica 181). The Hague: Mouton.

Oswalt, Robert L. 1979. An exploration of the affinity of Wappo and some Hokan and Penutian languages. In James E. Redden (ed.), *Proceedings of the 1978 Hokan Languages Workshop held at University of California, San Diego, June 27-29, 1978*, 56-71. (Occasional Papers on Linguistics 5). Carbondale, IL: Department of Linguistics, Southern Illinois University.

Pitkin, Harvey. 1985. *Wintu dictionary*. (University of California Publications in Linguistics 95). Berkeley, CA: University of California Press.

Press, Margaret L. 1979. *Chemehuevi, a grammar and lexicon*. (University of California Publications in Linguistics 92). Berkeley, CA: University of California Press.

Radin, Paul. 1929. A grammar of the Wappo language. *University of California Publications in American Archaeology and Ethnology* 27. 1-194.

Sawyer, Jesse O. 1965. *English-Wappo vocabulary*. (University of California Publications in Linguistics 43). Berkeley, CA: University of California Press.

Sawyer, Jesse O. 1991. *Wappo studies*. (Ed.) Alice Shepherd & William W. Elmendorf. (Survey of California and Other Indian Languages Report 7). Berkeley, CA: Department of Linguistics, University of California, Berkeley.

Schlichter, Alice. 1981. *Wintu dictionary*. (Survey of California and Other Indian Languages Report 2). Berkeley, CA: Department of Linguistics, University of California, Berkeley.

Schlichter, Alice. 1985. The Yukian language family. Berkeley, CA: University of California PhD dissertation.

Seiler, Hansjakob. 1977. *Cahuilla grammar*. Banning, CA: Malki Museum Press.

Seiler, Hansjakob. 1979. *Cahuilla dictionary*. Banning, CA: Malki Museum Press.

Shaterian, Alan William. 1983. Phonology and dictionary of Yavapai. Berkeley, CA: University of California PhD dissertation.

Shaul, David Leedom. 1988. Esselen: Utian onomastics. In William Shipley (ed.), *In honor of Mary Haas: From the Haas Festival Conference on Native American Linguistics*, 693-704. Berlin: Mouton de Gruyter.

Shaul, David Leedom. 1995. The Huelel (Esselen) language. *International Journal of American Linguistics* 61(2). 191-239.

Shepherd, Alice. 2006. *Proto-Wintun*. (University of California Publications in Linguistics 137). Berkeley, CA: University of California Press.

Shipley, William. 1957. Some Yukian-Penutian lexical resemblances. *International Journal of American Linguistics* 23(4). 269-274.

Shipley, William. 1961. Maidu and Nisenan: A binary survey. *International Journal of American Linguistics* 27(1). 46-51.

Shipley, William. 1963. *Maidu texts and dictionary*. (University of California Publications in Linguistics 33). Berkeley, CA: University of California Press.

Snapp, Allen, John Anderson & Joy Anderson. 1982. Northern Paiute. In Ronald W. Langacker (ed.), *Studies in Uto-Aztecan grammar, vol. 3: Uto-Aztecan grammatical sketches*. (Summer Institute of Linguistics Publications in Linguistics 56). Dallas, TX: Summer Institute of Linguistics.

Stubbs, Brian Darrell. 2008. *Uto-Aztecan: A comparative vocabulary*. Yorba Linda, CA: Shumway Family History Services.

Thompson, Sandra A, Joseph Sung-Yul Park & Charles N. Li. 2006. *A reference grammar of Wappo*. (University of California Publications in Linguistics 138). Berkeley, CA: University of California Press.

Thornes, Timothy Jon. 2003. A Northern Paiute grammar with texts. Eugene, OR: University of Oregon PhD dissertation.

Turner, Katherine. 1980. The reconstituted phonemes of Salinan. *Journal of California and Great Basin Anthropology* 2. 53-92.

Turner, Katherine. 1983. Areal and genetic linguistic affiliations of the Salinan. *Kansas Working Papers in Linguistics* 8(2). (Studies in Native American Languages 2). 215-246.

Uldall, Hans Jørgen & William Shipley. 1966. *Nisenan texts and dictionary*. (University of California Publications in Linguistics 46). Berkeley, CA: University of California Press.

Ultan, Russell. 1964. Proto-Maidun phonology. *International Journal of American Linguistics* 30(4). 355-370.

Vihman, Eero. 1976. On pitch accent in Northern Pomo. In Margaret Langdon & Shirley Silver (eds.), *Hokan Studies: Papers from the First Conference on Hokan Languages held in San Diego, California, April 23-25, 1970*, 77-86. (Janua Linguarum: Series Practica 181). The Hague: Mouton.

Voegelin, Charles F. 1935. Tübatulabal grammar. *University of California Publications in American Archaeology and Ethnology* 34(2). 55-189.

Voegelin, Erminie W. 1938. Tübatulabal ethnography. *Anthropological Records* 2(1). 1-90.

Warner, Natasha. n.d. Mutsun lexical database. ms.

Wash, Suzanne. 2001. Adverbial clauses in Barbareño Chumash narrative discourse. Santa Barbara, CA: University of California PhD dissertation.

Washo Project, The. 2008. Online Dictionary. http://washo.uchicago.edu/dictionary/dictionary.php (12 March, 2010).

Whistler, Kenneth W. & Victor Golla. 1986. Proto-Yokuts reconsidered. *International Journal of American Linguistics* 52(4). 317-358.

Zigmond, Maurice L., Curtis G. Booth & Pamela Munro. 1990. *Kawaiisu: A grammar and dictionary with texts*. (Ed.) Pamela Munro. (University of California Publications in Linguistics 119). Berkeley, CA: University of California Press.

**Amazonia (SAM):**

| **Family** | **Subgroup** | **Language** | **Source** |
| --- | --- | --- | --- |
| Arawak (North branch) | Rio Negro | Baniwa | Ramirez 2001a |
|  |  | Tariana | Aikhenvald 2001 |
|  |  | Yucuna | Schauer et al. 2005 |
|  | Western | Resígaro | Allin 1979 |
| Nadahup | Hup-Yuhup | Hup | Epps 2008, fieldnotes; Ramirez 2006 |
|  |  | Yuhup | V. Martins 2005; Ospina 2002 |
|  |  | Dâw | S. Martins 2004; V. Martins 2005 |
|  |  | Nadëb | V. Martins 1999, 2005 |
| Tukanoan | East Tukanoan | Cubeo | Morse, Salser & Salser 1999 |
|  |  | Desano | Alemán, López & Miller 2000 |
|  |  | Tukano | Ramirez 1997; West 1980 |
|  |  | Kotiria (Wanano) | Stenzel forthcoming; Waltz 2007 |
|  | West Tukanoan | Orejon | Velie & Velie 2008 |
| Yanomami | Yanomami-Yanomam | Yanomam | Migliazza 1972; Smole 1976 |
|  |  | Yanomami | Mattei-Müller 2007; Migliazza 1972; Milliken, Albert & Gómez 1999; Ramirez 1994; Finkers 1986 |
|  |  | Ninam | Gómez 1990; Migliazza 1972; Swain 2007 |
|  |  | Sanuma | Migliazza 1972; Taylor 1972 |
| Isolate |  | Hodi | Guarisma & Coppens 1978; Mattei-Müller n.d.; Rodman & Rodman 2007 |
| Isolate |  | Huaorani | Author unknown 1959; Peeke 2007 |
| Guahiban |  | Sikuani | Huber & Reed 1992; Queixalós 1988 |
| Kakua-Nukak |  | Kakua | Bolaños fieldnotes |
|  |  | Nukak | Cabrera, Franky & Mahecha 1999; Huber & Reed 1992 |
| Tupi-Guarani |  | Nheengatu | Grenand & Ferreira 1989 |
| Carib | Venezuelan | Makiritare | Hall 1988, 2007 |
|  |  | Panare | Mattei-Müller 1994; Price 2007 |
|  |  | Macushi | Abbott & Foster 2007; Carson 1982 |
|  |  | Pemon | Armellada & Gutiérrez 1981; Edwards 1977, 1980; Mosonyi & Mosonyi 2000 |
|  | Guianan | Carijona | Durbin & Seijas 1973; Landaburu 1996; Robayo 2000a |

These data were systematically compared with data from 72 additional South American languages, in order to identify loans and determine lexical histories. The complete 204-word list was not available for all of these languages, some of which have had minimal documentation. The languages and sources consulted are the following:

- **Arawak** family: Achagua (Huber & Reed 1992; Meléndez 1998), Piapoco (Klumpp 1995), Kabiyari (Huber & Reed 1992; Ramirez 2001b), Warekena (Aikhenvald 1998), Yavitero (Mosonyi 1987), Bare (Aikhenvald 1995), Paresi (Brandão fieldnotes), Wapishana (Melville, Tracy & Williams 2007; Santos 2006), Mandawaka (Ramirez 2001b).
- **Arutani-Sape** family: Arutani (Migliazza 1978), Sape (Migliazza 1978).
- **Bora** family: Bora (Aschmann 1993, Thiesen & Thiesen 1998), Muinane (Aschmann 1993).
- **Carib** family: Akawaio (Edwards 1980), Ingarikó (Souza 2005), Mapoyo (Mattei-Müller 1975), Wai Wai (Hawkins 2007), Waimiri-Atroari (Bruno 2003), Yabarana (Méndez-Arocha 1959), Yukpa (Robayo 2000b; Vegamián 1978).
- **Chibchan** family: Muisca (González 1987, 2007).
- **Choco** family: Northern Emberá (Sara 2001).
- **Gê** family: Kaingáng (Wiesemann 1981).
- **Guahiban** family: Guayabero (Huber & Reed 1992), Macaguan (Buenaventura 1993; Huber & Reed 1992), Cuiva (Huber & Reed 1992), Playero (Huber & Reed 1992)
- **Jivaroan** family: Ashuar (Fast, Fast & Fast 1996), Huambisa (Jakway 1987), Aguaruna (Wipio 1996).
- **Panoan** family: Matses (Fleck 2003).
- **Peba-Yaguan** family: Yagua (Powlison & Powlison 2007; Powlison 1995).
- **Quechua** family: Southern Pastaza Quechua (Landerman 2008), Inga (Tandioy et al. 1997), Napo Lowland Quichua (Orr & Wrisley 1981), Ayacucho Quechua (Soto 1976).
- **Saliban** family: Piaroa (Krute 1989; Mosonyi 2000).
- **Tukanoan** family: Bara (Huber & Reed 1992), Barasano (Huber & Reed 1992), Carapana (Huber & Reed 1992; Metzger 2000), Macuna (Huber & Reed 1992; Smothermon & Smothermon 1993), Yuruti (Huber & Reed 1992), Piratapuyo (Huber & Reed 1992), Siriano (Brandrup 1980), Tanimuca (Gaviria & Azcárate 1979; Mountain 1978), Tatuyo (Huber & Reed 1992), Tuyuca (Barnes 2007; Tamayo 1988), Waimaja (Huber & Reed 1992), Secoya (Fierro 1991; Piaguaje et al. 1992), Koreguaje (Cook & Gralow 2001), Siona (Huber & Reed 1992; Key 2007; Wheeler 1987).
- **Tupi-Guarani** family: Cocama (Espinosa 1989; Faust 2008).
- **Witoto** family: Ocaina (Aschmann 1993; Leach 1969), Nipode (Aschmann 1993), Minica (Aschmann 1993), Murui (Aschmann 1993; Burtch 1983).
- **Zaparoan** family: Iquito (Michael, Beier & Sullón 2006).
- **Isolates/unclassified**: Aymara (Cotari, Mejía & Corrasco 1978), Puinave (Girón 2008; Richardson 2007), Cofan (Borman 1976), Candoshi (Tuggy 2008), Taushiro (Alicea 1975), Maku (Migliazza 1978), Páez (Castillo 1877; Gerdel 2007; Slocum & Gerdel 1983), Ticuna (Anderson 1962), Urarina (Olawsky 2006), Camsa (Monguí 1981), Warao (Romero-Figueroa 1997), Andoke (Landaburu 1979), Huaorani (Author unknown 1959; Peeke 2007), Pume (Mosonyi & Mosonyi 2000; Yu 1997), Tinigua (Tobar 2000).

Subgrouping of the larger Amazonian families is still under discussion. This study relies on the existing state-of-the-art classifications: For Arawak, see Aikhenvald (1999) and Payne (1991) (see also Michael 2009 and Facundes 2002); for Carib, see Meira (2006); for Tukanoan, see Chacon (2009).

Abbott, Miriam & Patrick Foster. 2007. Macushi. In Mary Ritchie Key & Bernard Comrie (eds.), *The intercontinental dictionary series*. http://lingweb.eva.mpg.de/cgi-bin/ids/ids.pl?com=simple_ browse&lg_id=173 (9 August, 2011).

Aikhenvald, Alexandra Y. 1995. *Bare*. (Languages of the World: Materials 100). München: Lincom Europa.

Aikhenvald, Alexandra Y. 1998. Warekena. In Desmond C. Derbyshire & Geoffrey K. Pullum (eds.), *Handbook of Amazonian languages*, vol. 4, 225-439. Berlin: Mouton de Gruyter.

Aikhenvald, Alexandra Y. 1999. The Arawak language family. In R. M. W. Dixon & Alexandra Y. Aikhenvald (eds.), *The Amazonian languages*, 65-106. (Cambridge Language Surveys). Cambridge: Cambridge University Press.

Aikhenvald, Alexandra Y. 2001. Dicionário tariana-português / português-tariana. *Boletim do Museu Paraense Emílio Goeldi* 17(1). (Série Antropologia).

Alemán M., Tulio, Reinaldo López H. & Marion Miller. 2000. *Wirã, peamasa ya wererituri (desano-español): Diccionario bilingüe de 896 palabras*. Bogotá: Editorial Alberto Lleras Camargo.

Alicea Ortiz, Neftalí. 1975. *Vocabulario taushiro*. (Datos Etno-Lingüísticos 22). Lima: Instituto Lingüístico de Verano. http://www.sil.org/americas/peru/pubs/del22.pdf (9 August, 2011).

Allin, Trevor R. 1979. *Vocabulario resígaro*. (Documento De Trabajo 16). Yarinacocha, Peru: Instituto Lingüístico de Verano. http://www.sil.org/americas/peru/pubs/dt16.pdf (9 August, 2011).

Anderson, Doris. 1962. *Conversational Ticuna*. Yarinacocha, Peru: Instituto Lingüístico de Verano. http://www.sil.org/americas/peru/pubs/acd-convrsticuna.pdf (9 August, 2011).

Armellada, Cesáreo de & Mariano Gutiérrez Salazar. 1981. *Diccionario pemón: Pemón-castellano, castellano-pemón*. Caracas: CORPOVEN.

Aschmann, Richard P. 1993. *Proto Witotoan*. (Summer Institute of Linguistics and the University of Texas at Arlington Publications in Linguistics 114). Dallas, TX: Summer Institute of Linguistics.

Author unknown. 1959. *Estudios acerca de las lenguas huarani (auca), shimigae y zapara*. Quito: Instituto Lingüístico de Verano.

Barnes, Janet. 2007. Tuyuca. In Mary Ritchie Key & Bernard Comrie (eds.), *The intercontinental dictionary series*. http://lingweb.eva.mpg.de/cgi-bin/ids/ids.pl?com=simple_browse&lg_id= 257 (9 August, 2011).

Borman, M. B. 1976. *Vocabulario cofán: Cofán-castellano, castellano-cofán*. (Serie de vocabularios indígenas Mariano Silva y Aceves 19). Quito: Instituto Lingüístico de Verano.

Brandrup, Beverly Ann. 1980. *Vocabulario siriano y español*. Bogotá: Instituto Lingüístico de Verano.

Bruno, Ana Carla. 2003. Waimiri Atroari grammar: Some phonological, morphological, and syntactic aspects. Tuscon, AZ: University of Arizona PhD dissertation.

Buenaventura V., Edgar. 1993. *Observaciones preliminares acerca del idioma macaguan: Apuntes culturales, fonología, apuntes gramaticales, vocabulario macaguan-español*. (Ed.) Stephen H. Levinsohn. Bogotá: Instituto Lingüístico de Verano. http://www.sil.org/americas/colombia/ pubs/32989.pdf (9 August, 2011).

Burtch, Shirley. 1983. *Diccionario huitoto murui*. (Serie lingüística peruana 20). Yarinacocha, Peru: Instituto Lingüístico de Verano. http://www.sil.org/americas/peru/pubs/slp20-1.pdf, http://www.sil.org/americas/peru/pubs/slp20-2.pdf (9 August, 2011).

Cabrera Becerra, Gabriel, Carlos E Franky Calvo & Dany Mahecha Rubio. 1999. *Los nĩkak, nómadas de la Amazonia colombiana*. Bogotá: Editorial Universidad Nacional.

Carson, Neusa M. 1982. Phonology and morphosyntax of Macuxi (Carib). Lawrence, KS: University of Kansas PhD dissertation.

Castillo y Orozco, Eugenio del. 1877. *Vocabulario páez-castellano: Catecismo, nociones gramaticales i dos pláticas*. (Collection linguistique américaine 2). Paris: Maisonneuve.

Chacon, Thiago. 2009. Preliminary investigation of Proto-Tukanoan stops and Tukanoan family classification. Paper presented at the 53rd International Congress of Americanists, Mexico City, July 22.

Cook, Dorothy M. & Frances L. Gralow. 2001. *Diccionario bilingüe: Koreguaje-español, español-koreguaje*. Bogotá: Editorial Alberto Lleras Camargo.

Cotari, Daniel, Jaime Mejía & Víctor Carrasco. 1978. *Diccionario aymara-castellano, castellano-aymara*. Cochabamba, Bolivia: Instituto de Idiomas, Padres de Maryknoll.

Durbin, Marshall & Haydée Seijas. 1973. Proto Hianacoto: Guaque-Carijona-Hianacoto Umaua. *International Journal of American Linguistics* 39(1). 22-31.

Edwards, Walter F. 1977. Some aspects of the grammar and phonology of Akawaio and Arekuna. In Walter F. Edwards (ed.), *An introduction to the Akawaio and Arekuna peoples*. Georgetown, Guyana: Amerindian Languages Project, University of Guyana.

Edwards, Walter F. (ed.). 1980. *A short grammar and dictionary of the Akawaio and Arekuna languages of Guyana*. Georgetown, Guyana: Amerindian Languages Project, University of Guyana.

Epps, Patience. 2008. *A grammar of Hup*. (Mouton Grammar Library 43). Berlin: Mouton de Gruyter.

Espinosa, Lucas. 1989. *Breve diccionario analítico castellano-tupí del Perú. Sección cocama*. (Serie palabras 2). Iquitos, Peru: Ediciones C.E.T.A.

Facundes, Sidney da Silva. 2002. Historical linguistics and its contribution to improving knowledge of Arawak. In Jonathan D. Hill & Fernando Santos-Granero (eds.), *Comparative Arawakan histories: Rethinking language family and culture area in Amazonia*, 74-98. Urbana, IL: University of Illinois Press.

Fast Mowitz, Gerhard, Ruby Warkentin de Fast & Daniel Fast Warkentin. 1996. *Diccionario achuar-shiwiar - castellano*. (Serie lingüística peruana 36). Yarinacocha, Peru: Instituto Lingüístico de Verano. http://www.sil.org/americas/peru/pubs/slp36.pdf (9 August, 2011).

Faust W., Norma. 2008. *Gramática cocama: Lecciones para el aprendizaje del idioma cocama*. 3rd ed. (Serie lingüística peruana 6). Lima: Instituto Lingüístico de Verano. http://www.sil.org/ americas/peru/pubs/slp06.pdf (9 August, 2011).

Fierro, Gustavo A. 1991. *Listas comparativas de palabras en diez idiomas autóctonos ecuatorianos*. (Cuadernos etnolingüísticos 13). Quito: Instituto Lingüístico de Verano. http://www.sil.org/ acpub/ repository/30837.pdf (9 August, 2011).

Finkers, Juan. 1986. *Los yanomami y su sistema alimenticio: Yanomami nɨɨ pë*. Puerto Ayacucho, Venezuela: Vicariato Apostólico de Puerto Ayacucho.

Fleck, David William. 2003. A grammar of Matses. Houston, TX: Rice University PhD dissertation.

Gaviria T., Sofía Victoria & Luis José Azcárate G. 1979. *Fonología y lexicología de la lengua tanimuca: Semestre de campo*. (Lecturas en teoría y práctica en etnolingüística 1). Bogotá: Universidad de los Andes, Facultad de Artes y Ciencias, Departamento de Antropología.

Gerdel, Florence L. 2007. Paéz. In Mary Ritchie Key & Bernard Comrie (eds.), *The intercontinental dictionary series*. http://lingweb.eva.mpg.de/cgi-bin/ids/ids.pl?com=simple_browse&lg_id= 244 (9 August, 2011).

Girón Higuita, Jesús Mario. 2008. Una gramática del wãńsöjöt (puinave). Amsterdam: Vrije Universiteit Amsterdam PhD dissertation.

Gómez, Gale Goodwin. 1993. The Shiriana dialect of Yanam (northern Brazil). New York: Columbia University PhD dissertation.

González de Pérez, María Stella. 1987. *Diccionario y gramática chibcha: Manuscrito anónimo de la Biblioteca Nacional de Colombia*. (Biblioteca “Ezequiel Uricoechea” 1). Bogotá: Instituto Caro y Cuervo.

González de Pérez, María Stella. 2007. Muisca. In Mary Ritchie Key & Bernard Comrie (eds.), *The intercontinental dictionary series*. http://lingweb.eva.mpg.de/cgi-bin/ids/ids.pl?com=simple_ browse&lg_id=243 (9 August, 2011).

Grenand, Françoise & Epaminondas Henrique Ferreira. 1989. *Pequeno dicionário da língua geral*. (Série Amazonas cultura regional 6). Manaus, Brazil: Secretaria da Educação e Cultura, Coordenadoria de Assuntos Educacionais, Núcleo de Recursos Tecnológicos.

Guarisma Pinto, Virginia & Walter Coppens. 1978. Vocabulario hoti. *Antropológica* 49. 3-28.

Hall, Katherine. 1988. The morphosyntax of discourse in De’kwana Carib. St. Louis, MO: Washington University in St. Louis PhD dissertation.

Hawkins, Robert E. 2007. Wai Wai. In Mary Ritchie Key & Bernard Comrie (eds.), *The intercontinental dictionary series*. http://lingweb.eva.mpg.de/cgi-bin/ids/ids.pl?com=simple_ browse&lg_id=175 (9 August, 2011).

Huber, Randall Q. & Robert B. Reed. 1992. *Vocabulario comparativo: Palabras selectas de lenguas indígenas de Colombia (Comparative vocabulary: Selected words in indigenous languages of Colombia)*. Bogotá: Instituto Lingüístico de Verano. http://www.sil.org/americas/colombia/ pubs/32105.pdf (9 August, 2011).

Jakway, Martha A. 1987. *Vocabulario huambisa*. 2nd ed. (Serie lingüística peruana 24). Yarinacocha, Peru: Instituto Lingüístico de Verano. http://www.sil.org/americas/peru/pubs/slp24.pdf (9 August, 2011).

Key, Mary Ritchie. 2007. Siona. In Mary Ritchie Key & Bernard Comrie (eds.), *The intercontinental dictionary series*. http://lingweb.eva.mpg.de/cgi-bin/ids/ids.pl?com=simple_browse&lg_id= 256 (9 August, 2011).

Klumpp, Deloris A. Pharris de. 1995. *Vocabulario piapoco-español*. Bogotá: Instituto Lingüístico de Verano. http://www.sil.org/americas/colombia/pubs/VocPiapoco_36065.pdf (9 August, 2011).

Krute, Laurence Dana. 1989. Piaroa nominal morphosemantics. New York: Columbia University PhD dissertation.

Landaburu, Jon. 1979. *La langue des Andoke (Amazonie colombienne): Grammaire*. (Langues et civilisations à orale 36). Paris: Société d’études linguistiques et anthropologiques de France.

Landaburu, Jon (ed.). 1996. *Documentos sobre lenguas aborígenes de Colombia del archivo de Paul Rivet*. Bogotá: Ediciones Uniandes, Centro Colombiano de Estudios de Lenguas Aborígenes.

Landerman, Peter. 1973. *Vocabulario quechua del Pastaza*. (Serie lingüística peruana 8). Yarinacocha, Peru: Instituto Lingüístico de Verano. http://www.sil.org/americas/peru/pubs/ slp08.pdf (9 August, 2011).

Landin, David. 1983. *Dicionário e léxico: Karitiana/português*. Brasília: Summer Institute of Linguistics. http://www.sil.org/americas/brasil/publcns/dictgram/KTDic.pdf (9 August, 2011).

Leach, Ilo M. 1969. *Vocabulario ocaina*. (Serie lingüística peruana 4). Yarinacocha, Peru: Instituto Lingüístico de Verano. http://www.sil.org/americas/peru/pubs/slp04.pdf (9 August, 2011).

Martins, Silvana Andrade. 2004. Fonologia e gramática Dâw. Amsterdam: Vrije Universiteit Amsterdam PhD dissertation.

Martins, Valteir. 1999. Dicionário nadëb-português, português-nadëb. ms.

Martins, Valteir. 2005. Reconstrução fonológica do protomaku oriental. Amsterdam: Vrije Universiteit Amsterdam PhD dissertation.

Mattei-Müller, Marie-Claude. n.d. Hoti word list. ms.

Mattei-Müller, Marie-Claude. 1975. Vocabulario básico de la lengua mapoya. *Antropológica* 42. 57-77.

Mattei-Müller, Marie-Claude. 1994. *Diccionario ilustrado panare-español, índice español-panare: Un aporte al estudio de los Panares-E’ñepa*. Caracas: Comisión Nacional Quinto Centenario.

Mattei-Müller, Marie-Claude. 2007. *Lengua y cultura yanomamɨ: Diccionario ilustrado yanomamɨ-español, español-yanomamɨ*. Caracas: Gobierno Bolivariano de Venezuela, Ministerio del Poder Popular para la Cultura, Consejo Nacional de la Cultura.

Meira, Sergio. 2006. Cariban languages. In Keith Brown (ed.), *Encyclopedia of language & linguistics*, vol. 2, 199-203. 2nd ed. Oxford: Elsevier.

Meléndez L., Miguel Angel. 1998. *La lengua achagua: Estudio gramatical*. (Lenguas aborígenes de Colombia: Descripciones 11). Bogotá: Colciencias.

Melville, Colette, Frances V. Tracy & Olive Williams. 2007. Wapishana. In Mary Ritchie Key & Bernard Comrie (eds.), *The intercontinental dictionary series*. http://lingweb.eva.mpg.de/cgi-bin/ids/ids.pl?com=simple_browse&lg_id=266 (9 August, 2011).

Méndez-Arocha, Alberto. 1959. Un vocabulario yabarana con apuntes fonémicos. *Antropológica* 7. 63-84.

Metzger, Ronald G. 2000. *Marĩ yaye mena carapana (Yaia yaye mena español macãrĩca tuti): Diccionario de 1000 palabras, carapana-español*. Bogotá: Buena Semilla.

Michael, Lev. 2009. Review of Henri Ramirez, Línguas arawak da Amazônia setentrional: Comparação e descrição. *International Journal of American Linguistics* 75(3). 447-450.

Michael, Lev, Christine Beier & Karina Sullón Acosta. 2006. Diccionario bilingüe iquito - castellano y castellano - iquito. Cabeceras Aid Project, ms. http://www.cabeceras.org/ildp06_iqt-span_ 131206.pdf, http://www.cabeceras.org/ildp06_sp-iqt_131206_web.pdf (10 August, 2011).

Migliazza, Ernest C. 1972. Yanomama grammar and intelligibility. Bloomington, IN: Indiana University PhD dissertation.

Migliazza, Ernest C. 1978. Maku, Sape and Uruak languages: Current status and basic lexicon. *Anthropological Linguistics* 20(3). 133-140.

Milliken, William, Bruce Albert & Gale Goodwin Gómez. 1999. *Yanomami: A forest people*. London: Royal Botanic Gardens, Kew.

Monguí Sánchez, José Raúl. 1981. *La lengua kamentzá: Fonética, fonología, textos*. (Publicaciones del Instituto Caro y Cuervo 59). Bogotá: Instituto Caro y Cuervo.

Morse, Nancy L., Jay K. Salser & Neva F. West de Salser. 1999. *Diccionario ilustrado bilingüe: Cubeo-español, español-cubeo*. Bogotá: Editorial Alberto Lleras Camargo.

Mosonyi, Esteban Emilio. 2000. Elementos gramaticales del idioma piaroa. In María Stella González de Pérez & María Luisa Rodríguez de Montes (eds.), *Lenguas indígenas de Colombia: Una visión descriptiva*, 657-668. Bogotá: Instituto Caro y Cuervo.

Mosonyi, Esteban Emilio & Jorge Carlos Mosonyi. 2000. *Manual de lenguas indígenas de Venezuela*. (Serie orígenes 3). Caracas: Fundación Bigott.

Mosonyi, Jorge Carlos. 1987. El idioma yavitero: Ensayo de gramática y diccionario. Caracas: Unversidad Central de Venezuela PhD dissertation.

Mountain, Kathy. 1978. Lista de palabras Swadesh y Rowe. *Artículos en Lingüística y Campos Afines* 4. 1-57.

Olawsky, Knut J. 2006. *A grammar of Urarina*. (Mouton Grammar Library 37). Berlin: Mouton de Gruyter.

Orr, Carolyn & Betsy Wrisley. 1981. *Vocabulario quichua del oriente*. 2nd ed. (Serie de vocabularios indígenas del Ecuador). Quito: Instituto Lingüístico de Verano. http://sil.org/americas/peru/ pubs/34064.pdf (10 August, 2011).

Ospina Bozzi, Ana María. 2002. Les structures élémentaires du yuhup maku, langue de l’Amazonie colombienne: Morphologie et syntaxe. Paris: Université Paris 7 – Denis Diderot PhD dissertation.

Payne, David L. 1991. A classification of Maipuran (Arawakan) languages based on shared lexical retentions. In Desmond C. Derbyshire & Geoffrey K. Pullum (eds.), *Handbook of Amazonian languages*, vol. 3, 355-500. Berlin: Mouton de Gruyter.

Peeke, Colette. 2007. Waorani. In Mary Ritchie Key & Bernard Comrie (eds.), *The intercontinental dictionary series*. http://lingweb.eva.mpg.de/cgi-bin/ids/ids.pl?com=simple_browse&lg_id= 255 (10 August, 2011).

Piaguaje, Ramó, Elías Piaguaje, Orville E. Johnson & Mary Johnson. 1992. *Vocabulario secoya*. Quito: Instituto Lingüístico de Verano.

Politis, Gustavo G. 2007. *Nukak: Ethnoarcheology of an Amazonian people*. Walnut Creek, CA: Left Coast Press.

Powlison, Esther & Paul S. Powlison. 2007. Yagua. In Mary Ritchie Key & Bernard Comrie (eds.), *The intercontinental dictionary series*. http://lingweb.eva.mpg.de/cgi-bin/ids/ids.pl?com=simple_ browse&lg_id=260 (10 August, 2011).

Powlison, Paul S. 1995. *Nijya̠mi̠ niquejadamusiy ma̠y niquejadamuju̠, ma̠y niquejadamusiy nijya̠mi̠ niquejadamuju̠ (Diccionario yagua-castellano)*. (Serie lingüística peruana 35). Lima: Instituto Lingüístico de Verano. http://www.sil.org/americas/peru/pubs/slp35.pdf (10 August, 2011).

Price, Esther. 2007. Panare. In Mary Ritchie Key & Bernard Comrie (eds.), *The intercontinental dictionary series*. http://lingweb.eva.mpg.de/cgi-bin/ids/ids.pl?com=simple_browse&lg_id= 172 (10 August, 2011).

Queixalós, Francisco. 1988. *Diccionario sikuani-español*. (Lenguas aborígenes de Colombia 1). Bogotá: Universidad de los Andes, Centro Colombiano de Estudios en Lenguas Aborígenes.

Ramirez, Henri. 1994. Le parler yanomamɨ des xamatauteri. Aix-en-Provence, France: Université de Provence – Aix-Marseille 1 PhD dissertation.

Ramirez, Henri. 1997. *A fala tukano dos ye’pâ-masa*. Manaus, Brazil: Inspetoria Salesiana Missionária da Amazônia.

Ramirez, Henri. 2001a. *Dicionário da língua baniwa*. Manaus, Brazil: Editora da Universidade do Amazonas.

Ramirez, Henri. 2001b. *Línguas arawak da Amazônia setentrional: Comparação e descrição*. Manaus, Brazil: Editora da Universidade do Amazonas.

Richardson, Larry A. 2007. Puinave. In Mary Ritchie Key & Bernard Comrie (eds.), *The intercontinental dictionary series*. http://lingweb.eva.mpg.de/cgi-bin/ids/ids.pl?com=simple_ browse&lg_id=249 (10 August, 2011).

Robayo Moreno, Camilo Alberto. 2000a. Avance sobre morfología carijona. In María Stella González de Pérez & María Luisa Rodríguez de Montes (eds.), *Lenguas indígenas de Colombia: Una visión descriptiva*, 171-180. Bogotá: Instituto Caro y Cuervo.

Robayo Moreno, Camilo Alberto. 2000b. Introducción al estudio de la lengua yupo o yukpa. In María Stella González de Pérez & María Luisa Rodríguez de Montes (eds.), *Lenguas indígenas de Colombia: Una visión descriptiva*, 709-717. Bogotá: Instituto Caro y Cuervo.

Rodman, David & Sue Rodman. 2007. Yuwana. In Mary Ritchie Key & Bernard Comrie (eds.), *The intercontinental dictionary series*. http://lingweb.eva.mpg.de/cgi-bin/ids/ids.pl?com=simple_ browse&lg_id=250 (10 August, 2011).

Romero-Figueroa, Andrés. 1997. *A reference grammar of Warao*. (LINCOM Studies in Native American Linguistics). München: Lincom Europa.

Santos, Manoel Gomes dos. 2006. Uma gramatica do wapixana (aruak): Aspectos da fonologia, da morfologia e da sintaxe. Campinas, Brazil: Universidade Estadual de Campinas PhD dissertation.

Sara, Solomon. 2001. *A tri-lingual dictionary of Embera-Spanish-English*. (Languages of the World: Dictionaries 38). München: Lincom Europa.

Schauer, Stanley, Junia Schauer, Eladio Yucuna & Walter Yucuna. 2005. *Meke kemakánaka puráka’aloji: Wapura’akó chu, eyá karíwana chu (Diccionario bilingüe: Yukuna - español, español - yukuna)*. Bogotá: Editorial Fundación para el Desarrollo de los Pueblos Marginados. http://www.sil.org/americas/colombia/pubs/abstract.asp?id=45802 (10 August, 2011).

Slocum, Marianna C. & Florence L. Gerdel. 1983. *Diccionario páez-español, español-páez*. Lomalinda, Colombia: Editorial Townsend.

Smole, William J. 1976. *The Yanoama Indians: A cultural geography*. (Texas Pan American Series). Austin, TX: University of Texas Press.

Smothermon, Jeffrey R. & Josephine H. Smothermon. 1993. *Masa ye, gawa ye rãca ãmara tuti: Macuna-español, diccionario de 850 palabras*. Bogotá: Editorial Alberto Lleras Camargo.

Soto Ruiz, Clodoaldo. 1976. *Diccionario quechua, ayacucho-chanca*. Lima: Ministerio de Educación.

Stenzel, Kristine. forthcoming. *A reference grammar of Kotiria (Wanano)*. Lincoln, NE: University of Nebraska Press.

Swain, Carol. 2007. Ninam (Shirishana). In Mary Ritchie Key & Bernard Comrie (eds.), *The intercontinental dictionary series*. http://lingweb.eva.mpg.de/cgi-bin/ids/ids.pl?com=simple_ browse&lg_id=252 (10 August, 2011).

Tamayo L., César. 1988. *Mi primer diccionario: Español-tuyuca, tuyuca-español*. s.l.: s.n.

Tandioy Janasoy, Francisco, Stephen H. Levinsohn, Domingo Tandioy Chasoy & Alonso Maffla Bilbao. 1997. Diccionario inga (edición interina en el nuevo alfabeto). ms. http://www.sil.org/ americas/colombia/pubs/IngaDicc_2Edms_48151.pdf (10 August, 2011).

Taylor, Kenneth Iain. 1972. Sanuma (Yanoama) food prohibitions: The multiple classification of society and fauna. Madison, WI: University of Wisconsin PhD dissertation.

Thiesen, Wesley & Eva Thiesen. 1998. *Diccionario bora-castellano, castellano-bora*. (Serie lingüística peruana 46). Lima: Instituto Lingüístico de Verano. http://www.sil.org/americas/peru/pubs/ slp46.pdf (10 August, 2011).

Tobar Ortiz, Nubia. 2000. La lengua tinigua: Anotaciones fonológicas y morfológicas. In María Stella González de Pérez & María Luisa Rodríguez de Montes (eds.), *Lenguas indígenas de Colombia: Una visión descriptiva*, 669-679. Bogotá: Instituto Caro y Cuervo.

Tuggy, John C. 1966. *Vocabulario candoshi de Loreto*. (Serie Linguistica Peruana 2). Yarinacocha, Peru: Instituto Lingüístico de Verano. http://www.sil.org/americas/peru/pubs/slp02.pdf (10 August, 2011).

Vegamián, Félix María de. 1978. *Diccionario ilustrado yupa español, español yupa: Con onomástica y apuntaciones gramaticales*. Caracas: Formateca.

Velie, Daniel & Virginia Velie. 1981. *Vocabulario orejón*. (Serie lingüística peruana 16). Yarinacocha, Peru: Instituto Lingüístico de Verano. http://www.sil.org/americas/peru/pubs/slp16.pdf (10 August, 2011).

Waltz, Nathan E. 2007. *Diccionario bilingüe: Wanano o guanano—español, español—wanano o guanano*. Bogotá: Editorial Fundación para el Desarrollo de los Pueblos Marginados. http://www.sil.org/americas/colombia/pubs/WananoDict_49346.pdf (10 August, 2011).

West, Birdie. 1980. *Gramática popular del tucano*. Bogotá: Instituto Lingüístico de Verano. http://www.sil.org/americas/colombia/pubs/21835.pdf (10 August, 2011).

Wheeler, Alvaro. 1987. *Gantëya bain (El pueblo siona del río Putumayo, Colombia)*. Bogotá: Instituto Lingüístico de Verano. http://www.sil.org/americas/colombia/pubs/22017_vol1.pdf, http://www.sil.org/americas/colombia/pubs/22017_vol2.pdf (10 August, 2011).

Wiesemann, Ursula. 1981. *Dicionário kaingáng-português, português-kaingáng*. 2nd ed. Brasilia: Summer Institute of Linguistics.

Wipio Deicat, Gerardo. 1996. *Diccionario aguaruna-castellano, castellano-aguaruna*. (Ed.) Alejandro Paati Antunce & Martha Jakway. (Serie lingüística peruana 39). Lima: Instituto Lingüístico de Verano. http://www.sil.org/americas/peru/pubs/slp39.pdf (10 August, 2011).

Yu, Pei-Lin. 1997. *Hungry lightning: Notes of a woman anthropologist in Venezuela*. Albuquerque, NM: University of New Mexico Press.
