# Supplementary material for: Does Lateral Transmission Obscure Inheritance in Hunter-Gatherer Languages?
Source: PLoS One. 2011 Sep 27;6(9):e25195. doi: 10.1371/journal.pone.0025195 (PMC3181316; doi:10.1371/journal.pone.0025195)
Supplement: Table S2 — Language data: Etymological counts. (DOC) [file pone.0025195.s004.doc]

**Table S2. Language Data: Etymological Counts**

The following table provides the raw information for each case study area; each language was coded for a number of loan types (Loan Max is the sum of Dir. unk (loans which can be identified as such in two or more languages but where the direction of loan is unknown), Loan, Doubtful Loan and Loan into Proto-Language); missing data is identified; Inherited, Unique, words which are inherited but with semantic shift, and ‘other’ items (mostly words of unknown provenance but where exhaustive searches for etymology were not possible). Loan Min % and Loan Max % are percentages of the list that are loaned, excluding missing data.

| **Language** | **Loan Max** | **Dir. unk** | **Loan** | **Doubtful** | **Proto-Loan** | **Missing** | **Inherited** | **Unique** | **Sem. Shift** | **Other** | **Loan-Min %** | **Loan-Max %** |
| --- | --- | --- | --- | --- | --- | --- | --- | --- | --- | --- | --- | --- |
| Baniwa | 2 | 2 | 0 | 0 | 0 | 23 | 132 | 39 | 3 | 5 | 0.00 | 0.01 |
| Resígaro | 10 | 3 | 4 | 3 | 0 | 77 | 69 | 36 | 1 | 11 | 0.03 | 0.08 |
| Tariana | 7 | 2 | 3 | 2 | 0 | 43 | 112 | 34 | 2 | 6 | 0.02 | 0.04 |
| Yucuna | 3 | 2 | 0 | 1 | 0 | 37 | 92 | 65 | 0 | 7 | 0.00 | 0.02 |
| Dâw | 3 | 0 | 1 | 1 | 1 | 21 | 119 | 53 | 5 | 3 | 0.01 | 0.02 |
| Hup | 5 | 0 | 3 | 1 | 1 | 12 | 129 | 54 | 1 | 3 | 0.02 | 0.03 |
| Nadëb | 5 | 0 | 2 | 2 | 1 | 18 | 108 | 64 | 1 | 8 | 0.01 | 0.03 |
| Yuhup | 5 | 0 | 3 | 1 | 1 | 40 | 124 | 30 | 4 | 1 | 0.02 | 0.03 |
| Cubeo | 3 | 2 | 0 | 1 | 0 | 17 | 102 | 68 | 1 | 13 | 0.00 | 0.02 |
| Desano | 3 | 1 | 1 | 0 | 1 | 43 | 121 | 32 | 1 | 4 | 0.01 | 0.02 |
| Tukano | 5 | 1 | 0 | 0 | 4 | 42 | 135 | 16 | 0 | 6 | 0.00 | 0.03 |
| Wanano | 4 | 0 | 0 | 1 | 3 | 20 | 141 | 27 | 1 | 11 | 0.00 | 0.02 |
| Ninam | 2 | 0 | 0 | 0 | 2 | 28 | 149 | 25 | 0 | 0 | 0.00 | 0.01 |
| Sanuma | 5 | 0 | 0 | 2 | 3 | 32 | 131 | 36 | 0 | 0 | 0.00 | 0.03 |
| Yanomam | 2 | 0 | 0 | 1 | 1 | 31 | 163 | 8 | 0 | 0 | 0.00 | 0.01 |
| Yanomami | 3 | 0 | 0 | 2 | 1 | 10 | 137 | 54 | 0 | 0 | 0.00 | 0.02 |
| Hodi | 10 | 0 | 5 | 5 | 0 | 21 | 0 | 173 | 0 | 0 | 0.03 | 0.05 |
| Huaorani | 0 | 0 | 0 | 0 | 0 | 16 | 0 | 188 | 0 | 0 | 0.00 | 0.00 |
| Sikuani | 9 | 3 | 1 | 3 | 2 | 141 | 23 | 10 | 0 | 21 | 0.02 | 0.14 |
| Kakua | 15 | 3 | 7 | 2 | 3 | 30 | 82 | 57 | 1 | 19 | 0.04 | 0.09 |
| Nukak | 10 | 3 | 3 | 1 | 3 | 48 | 84 | 60 | 0 | 2 | 0.02 | 0.06 |
| Nheengatu | 6 | 3 | 3 | 0 | 0 | 39 | 41 | 1 | 1 | 116 | 0.02 | 0.04 |
| Makiritare | 0 | 0 | 0 | 0 | 0 | 61 | 84 | 46 | 0 | 13 | 0.00 | 0.00 |
| Panare | 4 | 3 | 0 | 1 | 0 | 10 | 100 | 61 | 1 | 28 | 0.00 | 0.02 |
| Carijona | 5 | 1 | 2 | 2 | 0 | 40 | 94 | 48 | 0 | 17 | 0.01 | 0.03 |
| Macushi | 4 | 3 | 0 | 0 | 1 | 23 | 118 | 46 | 2 | 11 | 0.00 | 0.02 |
| Orejon | 4 | 1 | 1 | 0 | 2 | 27 | 120 | 26 | 0 | 27 | 0.01 | 0.02 |
| Bardi | 6 | 0 | 4 | 1 | 1 | 4 | 176 | 11 | 1 | 6 | 0.02 | 0.03 |
| Nyulnyul | 4 | 0 | 2 | 0 | 2 | 12 | 173 | 12 | 3 | 0 | 0.01 | 0.02 |
| Nimanburru | 1 | 0 | 0 | 0 | 1 | 46 | 144 | 11 | 2 | 0 | 0.00 | 0.01 |
| Ngumbarl | 6 | 0 | 2 | 2 | 2 | 37 | 124 | 36 | 1 | 0 | 0.01 | 0.04 |
| Yawuru | 16 | 0 | 11 | 3 | 2 | 7 | 137 | 22 | 9 | 13 | 0.06 | 0.08 |
| Nyikina | 13 | 0 | 5 | 3 | 5 | 16 | 143 | 19 | 3 | 10 | 0.03 | 0.07 |
| Warrwa | 5 | 0 | 2 | 0 | 3 | 58 | 116 | 14 | 3 | 8 | 0.01 | 0.03 |
| Mangala | 34 | 0 | 23 | 10 | 1 | 11 | 75 | 79 | 5 | 0 | 0.13 | 0.18 |
| Northern Mangarla | 29 | 0 | 23 | 6 | 0 | 59 | 59 | 57 | 0 | 0 | 0.17 | 0.20 |
| Northern Nyangumarta | 18 | 2 | 10 | 3 | 3 | 22 | 127 | 37 | 0 | 0 | 0.06 | 0.10 |
| Nyangumarta | 19 | 1 | 13 | 4 | 1 | 18 | 113 | 44 | 0 | 10 | 0.07 | 0.10 |
| Karajarri | 36 | 1 | 29 | 6 | 0 | 14 | 116 | 35 | 1 | 2 | 0.16 | 0.19 |
| Bunuba | 31 | 0 | 17 | 14 | 0 | 23 | 52 | 98 | 0 | 0 | 0.10 | 0.17 |
| Gooniyandi | 71 | 0 | 56 | 15 | 0 | 39 | 56 | 32 | 0 | 6 | 0.37 | 0.43 |
| Gajirrabeng | 14 | 0 | 14 | 0 | 0 | 14 | 97 | 79 | 0 | 0 | 0.07 | 0.07 |
| Miriwoong | 16 | 0 | 16 | 0 | 0 | 24 | 101 | 63 | 0 | 0 | 0.09 | 0.09 |
| Kija | 27 | 0 | 27 | 0 | 0 | 11 | 100 | 66 | 0 | 0 | 0.14 | 0.14 |
| Walmajarri | 32 | 0 | 26 | 6 | 0 | 3 | 155 | 14 | 0 | 0 | 0.13 | 0.16 |
| Mudburra | 82 | 0 | 75 | 7 | 0 | 8 | 67 | 47 | 0 | 0 | 0.40 | 0.42 |
| Gurindji | 98 | 0 | 98 | 0 | 0 | 1 | 85 | 19 | 0 | 1 | 0.48 | 0.48 |
| Jaru | 37 | 0 | 31 | 6 | 0 | 12 | 111 | 29 | 15 | 0 | 0.17 | 0.19 |
| Djapu | 5 | 1 | 4 | 0 | 0 | 55 | 62 | 68 | 2 | 12 | 0.03 | 0.03 |
| Djinang | 11 | 2 | 7 | 2 | 0 | 7 | 61 | 119 | 4 | 2 | 0.04 | 0.06 |
| Dhangu | 9 | 0 | 6 | 3 | 0 | 30 | 85 | 76 | 0 | 4 | 0.04 | 0.05 |
| Dhuwala (Gupapuyngu) | 7 | 1 | 5 | 1 | 0 | 29 | 73 | 95 | 0 | 0 | 0.03 | 0.04 |
| Yan-nhangu | 12 | 0 | 10 | 2 | 0 | 27 | 90 | 72 | 1 | 2 | 0.06 | 0.07 |
| Ritharrngu | 40 | 1 | 38 | 0 | 1 | 13 | 106 | 24 | 5 | 16 | 0.20 | 0.21 |
| Burarra | 32 | 3 | 22 | 7 | 0 | 20 | 7 | 138 | 0 | 7 | 0.13 | 0.17 |
| Ngandi | 27 | 2 | 25 | 0 | 0 | 32 | 53 | 58 | 0 | 34 | 0.15 | 0.16 |
| Nunggubuyu | 13 | 0 | 11 | 2 | 0 | 47 | 81 | 63 | 0 | 0 | 0.07 | 0.08 |
| Yandruwandha | 10 | 6 | 3 | 1 | 0 | 6 | 123 | 62 | 3 | 0 | 0.02 | 0.05 |
| Mount Freeling Diyari | 4 | 2 | 2 | 0 | 0 | 75 | 71 | 51 | 2 | 1 | 0.02 | 0.03 |
| Arabana | 27 | 9 | 9 | 9 | 0 | 11 | 83 | 76 | 5 | 2 | 0.05 | 0.14 |
| Diyari | 18 | 8 | 8 | 2 | 0 | 7 | 139 | 36 | 4 | 0 | 0.04 | 0.09 |
| Pitta-Pitta | 11 | 7 | 3 | 1 | 0 | 31 | 90 | 69 | 3 | 0 | 0.02 | 0.06 |
| Wangkayutyuru | 11 | 4 | 6 | 1 | 0 | 36 | 71 | 82 | 4 | 0 | 0.04 | 0.07 |
| DiyariREU | 28 | 6 | 22 | 0 | 0 | 82 | 67 | 25 | 2 | 0 | 0.19 | 0.23 |
| Mithaka | 8 | 4 | 3 | 1 | 0 | 75 | 87 | 30 | 4 | 0 | 0.02 | 0.06 |
| Karuwali | 4 | 1 | 0 | 3 | 0 | 135 | 34 | 30 | 1 | 0 | 0.00 | 0.06 |
| Ngamini | 16 | 6 | 4 | 6 | 0 | 6 | 129 | 50 | 3 | 0 | 0.02 | 0.08 |
| Yarluyandi | 4 | 1 | 2 | 1 | 0 | 66 | 99 | 30 | 3 | 2 | 0.01 | 0.03 |
| Yawarrawarrka | 14 | 7 | 5 | 2 | 0 | 24 | 112 | 53 | 1 | 0 | 0.03 | 0.08 |
| Nhirrpi | 8 | 2 | 6 | 0 | 0 | 74 | 80 | 39 | 3 | 0 | 0.05 | 0.06 |
| Guwa | 9 | 5 | 4 | 0 | 0 | 122 | 43 | 30 | 0 | 0 | 0.05 | 0.11 |
| Yanda | 9 | 4 | 4 | 1 | 0 | 133 | 33 | 29 | 0 | 0 | 0.06 | 0.13 |
| Malyangapa | 45 | 20 | 18 | 7 | 0 | 64 | 45 | 46 | 3 | 1 | 0.16 | 0.32 |
| Wadikali | 2 | 0 | 1 | 1 | 0 | 178 | 18 | 4 | 1 | 1 | 0.04 | 0.08 |
| Yardliyawarra | 5 | 2 | 2 | 1 | 0 | 173 | 23 | 3 | 0 | 0 | 0.07 | 0.16 |
| Chumash Barbareno | 9 | 4 | 5 | 0 | 0 | 9 | 78 | 108 | 0 | 0 | 0.03 | 0.05 |
| Chumash Cruzeno | 9 | 4 | 5 | 0 | 0 | 71 | 57 | 67 | 0 | 0 | 0.04 | 0.07 |
| Chumash Ineseno | 11 | 5 | 6 | 0 | 0 | 30 | 129 | 2 | 0 | 32 | 0.04 | 0.06 |
| Chumash Obispeno | 13 | 4 | 9 | 0 | 0 | 28 | 80 | 83 | 0 | 0 | 0.05 | 0.07 |
| Chumash Ventureno | 13 | 4 | 9 | 0 | 0 | 8 | 70 | 113 | 0 | 0 | 0.05 | 0.07 |
| Esselen | 23 | 12 | 11 | 0 | 0 | 104 | 2 | 75 | 0 | 0 | 0.13 | 0.23 |
| Washo | 11 | 3 | 8 | 0 | 0 | 6 | 0 | 187 | 0 | 0 | 0.04 | 0.06 |
| Salinan | 17 | 6 | 11 | 0 | 0 | 15 | 1 | 171 | 0 | 0 | 0.06 | 0.09 |
| Seri Comcaac | 1 |  | 1 | 0 | 0 | 2 | 0 | 201 | 0 | 0 | 0.00 | 0.00 |
| Wappo | 57 | 17 | 30 | 0 | 10 | 3 | 42 | 1 | 0 | 101 | 0.17 | 0.28 |
| Yuki | 19 | 7 | 1 | 0 | 11 | 5 | 132 | 48 | 0 | 0 | 0.01 | 0.10 |
| Central Sierra Miwok | 10 | 2 | 4 | 0 | 4 | 24 | 154 | 16 | 0 | 0 | 0.02 | 0.06 |
| Maidu | 9 | 5 | 2 | 0 | 2 | 5 | 127 | 63 | 0 | 0 | 0.01 | 0.05 |
| Mutsun | 17 | 9 | 8 | 0 | 0 | 2 | 79 | 106 | 0 | 0 | 0.04 | 0.08 |
| Nisenan | 8 | 4 | 2 | 0 | 2 | 18 | 123 | 55 | 0 | 0 | 0.01 | 0.04 |
| Northern Sierra Miwok | 12 | 3 | 4 | 1 | 4 | 4 | 165 | 1 | 0 | 22 | 0.02 | 0.06 |
| Plains Miwok | 11 | 3 | 6 | 1 | 1 | 11 | 131 | 51 | 0 | 0 | 0.03 | 0.06 |
| Southern Sierra Miwok | 15 | 5 | 8 | 0 | 2 | 6 | 148 | 1 | 0 | 34 | 0.04 | 0.08 |
| Wintu | 22 | 13 | 3 | 0 | 6 | 1 | 143 | 38 | 0 | 0 | 0.02 | 0.11 |
| Yokuts | 19 | 11 | 2 | 1 | 5 | 23 | 138 | 23 | 0 | 1 | 0.01 | 0.10 |
| Yokuts Palewyami | 10 | 5 | 1 | 0 | 4 | 80 | 73 | 41 | 0 | 0 | 0.01 | 0.08 |
| Yokuts Yawdanchi | 10 | 4 | 0 | 0 | 6 | 42 | 124 | 28 | 0 | 0 | 0.00 | 0.06 |
| Lake Miwok | 28 | 5 | 23 | 0 | 0 | 3 | 86 | 87 | 0 | 0 | 0.12 | 0.14 |
| Cahuilla | 15 | 7 | 8 | 0 | 0 | 6 | 150 | 16 | 0 | 17 | 0.04 | 0.08 |
| Chemehuevi | 9 | 7 | 0 | 0 | 2 | 17 | 162 | 5 | 0 | 11 | 0.00 | 0.05 |
| Cupeno | 13 | 2 | 11 | 0 | 0 | 2 | 154 | 6 | 0 | 29 | 0.06 | 0.06 |
| Gabrielino | 7 | 1 | 3 | 0 | 3 | 28 | 123 | 5 | 0 | 41 | 0.02 | 0.04 |
| Kawaiisu | 13 | 9 | 3 | 0 | 1 | 6 | 134 | 50 | 0 | 1 | 0.02 | 0.07 |
| Kitanemuk | 20 | 6 | 10 | 0 | 4 | 3 | 154 | 7 | 0 | 20 | 0.05 | 0.10 |
| Luiseno | 12 | 5 | 5 | 0 | 2 | 2 | 146 | 5 | 0 | 39 | 0.03 | 0.06 |
| Northern Paiute Thornes | 10 | 2 | 4 | 1 | 3 | 12 | 146 | 35 | 0 | 1 | 0.02 | 0.05 |
| Serrano | 13 | 4 | 8 | 0 | 1 | 9 | 149 | 33 | 0 | 0 | 0.04 | 0.07 |
| Tubatulabal | 38 | 7 | 30 | 0 | 1 | 18 | 99 | 49 | 0 | 0 | 0.17 | 0.20 |
| Bankalachi Toloim | 30 | 0 | 28 | 2 | 0 | 91 | 60 | 23 | 0 | 0 | 0.25 | 0.27 |
| Tumpisa Shoshone | 9 | 6 | 1 | 0 | 2 | 3 | 151 | 41 | 0 | 0 | 0.01 | 0.04 |
| Western Mono | 17 | 7 | 10 | 0 | 0 | 6 | 156 | 6 | 0 | 19 | 0.05 | 0.09 |
| Cocopa | 7 | 3 | 2 | 0 | 2 | 8 | 130 | 1 | 0 | 58 | 0.01 | 0.04 |
| Iipay Aa | 7 | 2 | 3 | 0 | 2 | 12 | 103 | 81 | 0 | 1 | 0.02 | 0.04 |
| Kiliwa | 6 | 1 | 1 | 2 | 2 | 8 | 108 | 82 | 0 | 0 | 0.01 | 0.03 |
| Mojave | 13 | 4 | 5 | 3 | 1 | 5 | 132 | 54 | 0 | 0 | 0.03 | 0.07 |
| Yavapai | 6 | 3 | 2 | 0 | 1 | 5 | 121 | 72 | 0 | 0 | 0.01 | 0.03 |
| Yuma | 2 | 1 | 1 | 0 | 0 | 81 | 96 | 25 | 0 | 0 | 0.01 | 0.02 |
| Big Smokey Valley Shoshona | 4 | 3 | 0 | 0 | 1 | 13 | 168 | 5 | 3 | 11 | 0.00 | 0.02 |
| Comanche | 7 | 3 | 1 | 0 | 3 | 5 | 158 | 20 | 5 | 9 | 0.01 | 0.04 |
| Southern Paiute | 6 | 3 | 0 | 0 | 3 | 27 | 139 | 32 | 0 | 0 | 0.00 | 0.03 |
| Southern Ute | 8 | 3 | 1 | 0 | 4 | 7 | 158 | 31 | 0 | 0 | 0.01 | 0.04 |

| **Language** | **CSA** | **Food Prod** | **Size** | **Density** | **Mobility** | **Exo-gamy** |
| --- | --- | --- | --- | --- | --- | --- |
| Baniwa | SAM | AG | large | dense | sedentary | no |
| Resígaro | SAM | AG | medium | medium | sedentary | no |
| Tariana | SAM | AG | medium | medium | sedentary | yes |
| Yucuna | SAM | AG | medium | medium | sedentary | no |
| Dâw | SAM | HG | small | low | mobile | no |
| Hup | SAM | HG | medium | low | mobile | no |
| Nadëb | SAM | HG | small | low | mobile | no |
| Yuhup | SAM | HG | small | low | mobile | no |
| Cubeo | SAM | AG | large | medium | sedentary | no |
| Desano | SAM | AG | medium | medium | sedentary | yes |
| Tukano | SAM | AG | medium | medium | sedentary | yes |
| Wanano | SAM | AG | medium | medium | sedentary | yes |
| Ninam | SAM | HG | medium | medium | seasonal | no |
| Sanuma | SAM | HG | medium | medium | seasonal | no |
| Yanomam | SAM | HG | medium | medium | seasonal | no |
| Yanomami | SAM | HG | medium | medium | seasonal | no |
| Hodi | SAM | HG | small | low | mobile | no |
| Huaorani | SAM | HG | medium | low | mobile | no |
| Sikuani | SAM | HG | large | medium | mobile | no |
| Kakua | SAM | HG | small | low | mobile | no |
| Nukak | SAM | HG | small | low | mobile | no |
| Nheengatu | SAM | AG | large | dense | sedentary | no |
| Makiritare | SAM | AG | large | medium | sedentary | no |
| Panare | SAM | AG | medium | medium | sedentary | no |
| Carijona | SAM | AG | small | medium | sedentary | no |
| Macushi | SAM | AG | large | dense | sedentary | no |
| Orejon | SAM | HG | medium | medium | sedentary | no |
| Bardi | AUS | HG | large | medium | seasonal | no |
| Nyulnyul | AUS | HG | medium | medium | seasonal | no |
| Nimanburru | AUS | HG | small | dense | sedentary | no |
| Ngumbarl | AUS | HG | small | dense | sedentary | no |
| Yawuru | AUS | HG | large | medium | sedentary | yes |
| Nyikina | AUS | HG | large | medium | seasonal | yes |
| Warrwa | AUS | HG | small | medium | seasonal | yes |
| Mangala | AUS | HG | small | medium | mobile | no |
| Northern Mangarla | AUS | HG | small | medium | mobile | no |
| Northern Nyangumarta | AUS | HG | small | medium | mobile | no |
| Nyangumarta | AUS | HG | medium | low | mobile | no |
| Karajarri | AUS | HG | medium | low | mobile | yes |
| Bunuba | AUS | HG | medium | low | mobile | no |
| Gooniyandi | AUS | HG | small | low | mobile | yes |
| Gajirrabeng | AUS | HG | small | low | mobile | X |
| Miriwoong | AUS | HG | medium | low | mobile | X |
| Kija | AUS | HG | medium | low | mobile | X |
| Walmajarri | AUS | HG | large | low | mobile | X |
| Mudburra | AUS | HG | medium | low | mobile | X |
| Gurindji | AUS | HG | medium | low | mobile | no |
| Jaru | AUS | HG | medium | low | mobile | X |
| Djapu | AUS | HG | small | dense | mobile | yes |
| Djinang | AUS | HG | medium | dense | mobile | yes |
| Dhangu | AUS | HG | small | dense | mobile | yes |
| Dhuwala (Gupapuyngu) | AUS | HG | medium | dense | mobile | yes |
| Yan-nhangu | AUS | HG | small | dense | mobile | yes |
| Ritharrngu | AUS | HG | medium | dense | mobile | yes |
| Burarra | AUS | HG | large | dense | mobile | yes |
| Ngandi | AUS | HG | small | dense | mobile | yes |
| Nunggubuyu | AUS | HG | medium | dense | mobile | yes |
| Yandruwandha | AUS | HG | large | medium | mobile | X |
| Mount Freeling Diyari | AUS | HG | small | medium | mobile | yes |
| Arabana | AUS | HG | large | low | mobile | X |
| Diyari | AUS | HG | large | medium | mobile | yes |
| Pitta-Pitta | AUS | HG | medium | medium | mobile | X |
| Wangkayutyuru | AUS | HG | small | medium | mobile | X |
| DiyariREU | AUS | HG | large | medium | seasonal | yes |
| Mithaka | AUS | HG | small | low | mobile | X |
| Karuwali | AUS | HG | small | low | mobile | X |
| Ngamini | AUS | HG | small | medium | mobile | X |
| Yarluyandi | AUS | HG | small | medium | mobile | X |
| Yawarrawarrka | AUS | HG | small | medium | mobile | X |
| Nhirrpi | AUS | HG | small | medium | mobile | x |
| Guwa | AUS | HG | medium | low | mobile | X |
| Yanda | AUS | HG | medium | low | mobile | X |
| Malyangapa | AUS | HG | medium | medium | mobile | X |
| Wadikali | AUS | HG | small | low | mobile | X |
| Yardliyawarra | AUS | HG | small | medium | mobile | X |
| Chumash Barbareno | NAM | HG | large | dense | sedentary | no |
| Chumash Cruzeno | NAM | HG | small | dense | sedentary | no |
| Chumash Ineseno | NAM | HG | medium | dense | sedentary | no |
| Chumash Obispeno | NAM | HG | small | dense | sedentary | no |
| Chumash Ventureno | NAM | HG | large | dense | sedentary | no |
| Esselen | NAM | HG | small | medium | seasonal | no |
| Washo | NAM | HG | medium | dense | sedentary | no |
| Salinan | NAM | HG | medium | medium | seasonal | no |
| Seri Comcaac | NAM | HG | medium | low | mobile | no |
| Wappo | NAM | HG | small | medium | seasonal | no |
| Yuki | NAM | HG | large | medium | seasonal | no |
| Central Sierra Miwok | NAM | HG | large | medium | seasonal | no |
| Maidu | NAM | HG | medium | medium | sedentary | no |
| Mutsun | NAM | HG | medium | medium | seasonal | no |
| Nisenan | NAM | HG | medium | medium | sedentary | no |
| Northern Sierra Miwok | NAM | HG | large | medium | seasonal | no |
| Plains Miwok | NAM | HG | large | medium | seasonal | no |
| Southern Sierra Miwok | NAM | HG | large | medium | seasonal | no |
| Wintu | NAM | HG | large | dense | seasonal | no |
| Yokuts | NAM | HG | medium | medium | seasonal | no |
| Yokuts Palewyami | NAM | HG | small | medium | seasonal | no |
| Yokuts Yawdanchi | NAM | HG | small | medium | seasonal | no |
| Lake Miwok | NAM | HG | small | medium | seasonal | no |
| Cahuilla | NAM | AG | large | low | seasonal | no |
| Chemehuevi | NAM | HG | small | low | sedentary | no |
| Cupeno | NAM | HG | small | medium | sedentary | no |
| Gabrielino | NAM | HG | large | dense | sedentary | no |
| Kawaiisu | NAM | HG | small | low | mobile | no |
| Kitanemuk | NAM | HG | small | low | mobile | no |
| Luiseno | NAM | HG | large | medium | seasonal | no |
| Northern Paiute Thornes | NAM | HG | large | low | mobile | no |
| Serrano | NAM | HG | medium | low | seasonal | no |
| Tubatulabal | NAM | HG | medium | medium | seasonal | no |
| Bankalachi Toloim | NAM | HG | small | medium | seasonal | no |
| Tumpisa Shoshone | NAM | AG | small | low | mobile | no |
| Western Mono | NAM | HG | large | low | seasonal | no |
| Cocopa | NAM | AG | large | dense | sedentary | no |
| Iipay Aa | NAM | HG | medium | medium | seasonal | no |
| Kiliwa | NAM | HG | small | low | mobile | no |
| Mojave | NAM | AG | large | dense | sedentary | no |
| Yavapai | NAM | AG | medium | low | seasonal | no |
| Yuma | NAM | AG | large | dense | sedentary | no |
| Big Smokey Valley Shoshona | NAM | AG | small | low | mobile | no |
| Comanche | NAM | AG | large | low | mobile | no |
| Southern Paiute | NAM | AG | medium | low | mobile | no |
| Southern Ute | NAM | AG | medium | low | mobile | no |
