# Supplementary material for: Does Lateral Transmission Obscure Inheritance in Hunter-Gatherer Languages?
Source: PLoS One. 2011 Sep 27;6(9):e25195. doi: 10.1371/journal.pone.0025195 (PMC3181316; doi:10.1371/journal.pone.0025195)
Supplement: Table S3 — Basic vocabulary list. (DOC) [file pone.0025195.s005.doc]

**Table S3 204 item word list**

The following list is based on that used by Greenhill et al (2008) in the Austronesian Basic Vocabulary Database (<http://language.psy.auckland.ac.nz/austronesian/>). Items which also appear in the Swadesh 100 list (Swadesh 1971) are asterisked, and those additional words in the 200 list (see for example Crowley and Bowern 2010) are marked with $.)

| **RecordID** | **English** | **Semantic Field** |
| --- | --- | --- |
| 1 | above | LOCATION |
| 2 | again | GRAMMAR |
| 3 | *all | NUMBER |
| 4 | $and | GRAMMAR |
| 5 | ankle | BODY |
| 6 | ant | FAUNA/FLORA |
| 7 | armpit | BODY |
| 8 | *ash | ENVIRONMENT |
| 9 | $at | LOCATION |
| 10 | *back | BODY |
| 11 | $bad/evil | QUALITY |
| 12 | *belly | BODY |
| 13 | below | LOCATION |
| 14 | *big | QUALITY |
| 15 | *bird | FAUNA/FLORA |
| 16 | *bite | BODY |
| 17 | *black | COLOUR |
| 18 | *blood | BODY |
| 19 | $blow | BODY |
| 20 | blowfly/housefly | FAUNA/FLORA |
| 21 | boil/pimple | BODY |
| 22 | *bone | BODY |
| 23 | boomerang/throwing stick | MANUFACTURE |
| 24 | bottom grinding stone | MANUFACTURE |
| 25 | *breast | BODY |
| 26 | $breathe | BODY |
| 27 | *burn | ENVIRONMENT |
| 28 | chew | BODY |
| 29 | $child | HUMAN |
| 30 | climb | MOTION |
| 31 | *cloud | ENVIRONMENT |
| 32 | *cold | QUALITY |
| 33 | *come | MOTION |
| 34 | $cook | IMPACT |
| 35 | correct/true | QUALITY |
| 36 | $count | MENTAL |
| 37 | cry | MENTAL |
| 38 | $cut/hack | IMPACT |
| 39 | $day | TIME |
| 40 | *die/be dead | STATE |
| 41 | $dig | IMPACT |
| 42 | digging stick | MANUFACTURE |
| 43 | dingo/wolf | FAUNA/FLORA |
| 44 | $dirty | QUALITY |
| 45 | *dog | FAUNA/FLORA |
| 46 | dream | MENTAL |
| 47 | *drink | BODY |
| 48 | *dry | QUALITY |
| 49 | $dull/blunt | QUALITY |
| 50 | $dust | ENVIRONMENT |
| 51 | *ear | BODY |
| 52 | *earth/soil | ENVIRONMENT |
| 53 | *eat | BODY |
| 54 | *egg | FAUNA/FLORA |
| 55 | *eye | BODY |
| 56 | faeces | BODY |
| 57 | $fall | MOTION |
| 58 | $far | QUALITY |
| 59 | $fat/grease | BODY |
| 60 | $father | KINSHIP |
| 61 | $fear | MENTAL |
| 62 | *feather | FAUNA/FLORA |
| 63 | *fire | ENVIRONMENT |
| 64 | *fish | FAUNA/FLORA |
| 65 | $flow | MOTION |
| 66 | $flower | ENVIRONMENT |
| 67 | *fly | MOTION |
| 68 | $fog | ENVIRONMENT |
| 69 | *foot | BODY |
| 70 | $fruit | FAUNA/FLORA |
| 71 | *good | QUALITY |
| 72 | $grass | ENVIRONMENT |
| 73 | grow | STATE |
| 74 | *hair | BODY |
| 75 | *hand | BODY |
| 76 | $he/she | GRAMMAR |
| 77 | *head | BODY |
| 78 | *hear | MENTAL |
| 79 | $heavy | QUALITY |
| 80 | hide | MOTION |
| 81 | $hit | IMPACT |
| 82 | $hold | OTHER |
| 83 | house | MANUFACTURE |
| 84 | $how? | GRAMMAR |
| 85 | *I | GRAMMAR |
| 86 | $if | GRAMMAR |
| 87 | $in/inside | LOCATION |
| 88 | $intestines | BODY |
| 89 | itch | BODY |
| 90 | kangaroo/deer | FAUNA/FLORA |
| 91 | *kill | IMPACT |
| 92 | *know/be knowledgeable | MENTAL |
| 93 | $lake | ENVIRONMENT |
| 94 | $laugh | MENTAL |
| 95 | *leaf | ENVIRONMENT |
| 96 | $left | LOCATION |
| 97 | $leg/foot | BODY |
| 98 | *lie down | MOTION |
| 99 | lightning | ENVIRONMENT |
| 100 | $live/be alive | BODY |
| 101 | *liver | BODY |
| 102 | *long | QUALITY |
| 103 | *louse | FAUNA/FLORA |
| 104 | lung | BODY |
| 105 | *man/male | HUMAN |
| 106 | *meat/flesh | FAUNA/FLORA |
| 107 | *moon | ENVIRONMENT |
| 108 | mosquito | FAUNA/FLORA |
| 109 | $mother | KINSHIP |
| 110 | *mouth | BODY |
| 111 | *name | HUMAN |
| 112 | nape | BODY |
| 113 | $near/close | QUALITY |
| 114 | *neck | BODY |
| 115 | *new | QUALITY |
| 116 | *night | TIME |
| 117 | *no/not | GRAMMAR |
| 118 | *nose | BODY |
| 119 | $old | QUALITY |
| 120 | *one | NUMBER |
| 121 | open/uncover | OTHER |
| 122 | $other | GRAMMAR |
| 123 | painful/sick | BODY |
| 124 | *person/human being | HUMAN |
| 125 | pound/beat | IMPACT |
| 126 | *rain | ENVIRONMENT |
| 127 | *red | COLOUR |
| 128 | $right | LOCATION |
| 129 | *road/path | MANUFACTURE |
| 130 | *root | FAUNA/FLORA |
| 131 | $rope | MANUFACTURE |
| 132 | $rotten | QUALITY |
| 133 | *sand | ENVIRONMENT |
| 134 | *say | MENTAL |
| 135 | $scratch | IMPACT |
| 136 | *see | MENTAL |
| 137 | $sharp | QUALITY |
| 138 | $shoot | IMPACT |
| 139 | $short | QUALITY |
| 140 | shoulder | BODY |
| 141 | shy/ashamed | MENTAL |
| 142 | *sit | STATE |
| 143 | *skin | BODY |
| 144 | $sky | ENVIRONMENT |
| 145 | *sleep | BODY |
| 146 | *small | QUALITY |
| 147 | *smoke | ENVIRONMENT |
| 148 | $snake | FAUNA/FLORA |
| 149 | $sniff/smell | BODY |
| 150 | $spear | MANUFACTURE |
| 151 | spearthrower | MANUFACTURE |
| 152 | spider | FAUNA/FLORA |
| 153 | $spit | BODY |
| 154 | $split | IMPACT |
| 155 | $squeeze | IMPACT |
| 156 | $stab/pierce | IMPACT |
| 157 | *stand | STATE |
| 158 | *star | ENVIRONMENT |
| 159 | steal | OTHER |
| 160 | stick/wood | ENVIRONMENT |
| 161 | *stone | ENVIRONMENT |
| 162 | $suck | BODY |
| 163 | sweat | BODY |
| 164 | $swell | BODY |
| 165 | *swim | MOTION |
| 166 | *tail | BODY |
| 167 | *that | GRAMMAR |
| 168 | thatch/roof | MANUFACTURE |
| 169 | $they | GRAMMAR |
| 170 | $thick | QUALITY |
| 171 | $thin | QUALITY |
| 172 | $think | MENTAL |
| 173 | *this | GRAMMAR |
| 174 | $thou | GRAMMAR |
| 175 | $three | NUMBER |
| 176 | throat | BODY |
| 177 | $throw | IMPACT |
| 178 | thunder | ENVIRONMENT |
| 179 | $tie up/fasten | IMPACT |
| 180 | *tongue | BODY |
| 181 | *tooth | BODY |
| 182 | top grinding stone | MANUFACTURE |
| 183 | $turn | OTHER |
| 184 | *two | NUMBER |
| 185 | $vomit | BODY |
| 186 | *walk | MOTION |
| 187 | *water | ENVIRONMENT |
| 188 | we-excl [pl] | GRAMMAR |
| 189 | *we-incl [pl] | GRAMMAR |
| 190 | $wet | QUALITY |
| 191 | *what? | GRAMMAR |
| 192 | $when? | GRAMMAR |
| 193 | $where? | GRAMMAR |
| 194 | *white | COLOUR |
| 195 | *who? | GRAMMAR |
| 196 | $wife | KINSHIP |
| 197 | $wind | ENVIRONMENT |
| 198 | $wing | BODY |
| 199 | winnow | MANUFACTURE |
| 200 | *woman/female | HUMAN |
| 201 | $work | OTHER |
| 202 | yawn | BODY |
| 203 | *yellow | COLOUR |
| 204 | *you | GRAMMAR |
