# Supplementary material for: Does Lateral Transmission Obscure Inheritance in Hunter-Gatherer Languages?
Source: PLoS One. 2011 Sep 27;6(9):e25195. doi: 10.1371/journal.pone.0025195 (PMC3181316; doi:10.1371/journal.pone.0025195)
Supplement: Text S1 — Ethnographic atlas codes. (DOC) [file pone.0025195.s006.doc]

**Text S1. Ethnographic Atlas codes**

The following chart details the compatibility of the codes used in this paper with the *Ethnographic Atlas* (Murdock 1964-1980).

61. FIXITY OF SETTLEMENT =SEDENTISM

# of Code Descriptive

Cases # = Label

--- - -----

28 1 = Migratory =MOBILE

21 2 = Seminomadic- fixed then migratory =SEASONAL

6 3 = Rotating among 2+ fixed =SEASONAL

14 4 = Semisedentary- fixed core, some migratory =SEDENTARY

15 5 = Impermanent- periodically moved =SEDENTARY

102 6 = Permanent =SEDENTARY

63. COMMUNITY SIZE

1 . = Missing Data

28 1 = < 50 =SMALL

28 2 = 50-99 =SMALL

45 3 = 100-199 =MEDIUM

32 4 = 200-399 =MEDIUM

29 5 = 400-999 =MEDIUM

15 6 = 1,000-4,999 =LARGE

5 7 = 5,000-49,999 =LARGE

3 8 = > 50,000 =LARGE

64. POPULATION DENSITY

2 . = Missing Data

36 1 = < 1 person per 5 sq. mile =LOW

22 2 = 1 person per 1-5 sq. mile =LOW

25 3 = 1-5 persons per sq. mile =MEDIUM

27 4 = 1-25 persons per sq. mile =MEDIUM

34 5 = 26-100 persons per sq. mile =DENSE

20 6 = 101-500 persons per sq. mile =DENSE

20 7 = over 500 persons per sq. mile =DENSE
